# Tailoring confocal microscopy for real-time analysis of photosynthesis at single-cell resolution

**DOI:** 10.1016/j.crmeth.2023.100568

**Published:** 2023-08-28

**Authors:** Mattia Storti, Haythem Hsine, Clarisse Uwizeye, Olivier Bastien, Daniel P. Yee, Fabien Chevalier, Johan Decelle, Cécile Giustini, Daniel Béal, Gilles Curien, Giovanni Finazzi, Dimitri Tolleter

**Affiliations:** 1 Grenoble Alpes University, CNRS, CEA, INRAE, IRIG-LPCV, 38000 Grenoble, France; 2 Cell Biology and Biophysics Unit, European Molecular Biology Laboratory, 69117 Heidelberg, Germany; 3 JBeamBio, 17000 La Rochelle, France

**Keywords:** confocal microscopy, photoprotection, phytoplankton, non-vascular and vascular plants, light penetration, 3D reconstruction, fluorescence integration

## Abstract

Photoautotrophs’ environmental responses have been extensively studied at the organism and ecosystem level. However, less is known about their photosynthesis at the single-cell level. This information is needed to understand photosynthetic acclimation processes, as light changes as it penetrates cells, layers of cells, or organs. Furthermore, cells within the same tissue may behave differently, being at different developmental/physiological stages. Here, we describe an approach for single-cell and subcellular photophysiology based on the customization of confocal microscopy to assess chlorophyll fluorescence quenching by the saturation pulse method. We exploit this setup to (1) reassess the specialization of photosynthetic activities in developing tissues of non-vascular plants; (2) identify a specific subpopulation of phytoplankton cells in marine photosymbiosis, which consolidate energetic connections with their hosts; and (3) examine the link between light penetration and photoprotection responses inside the different tissues that constitute a plant leaf anatomy.

## Highlights

• Real-time assessment of chlorophyll fluorescence at confocal microscopy resolution
• Study of photosynthesis at single-cell resolution within a living tissue/organism
• Relationship between photosynthetic features and cell type or morphology
• Mathematical modeling of photosynthesis in a cell volume 3D space

## Motivation

Visualizing photosynthetic responses in 3D is essential for understanding most acclimation processes, as light changes within photosynthetic tissues as it penetrates the absorbing/diffusing layers of the cells. To achieve this goal, we developed an imaging workflow merging confocal microscopy and saturating pulse chlorophyll fluorescence detection. This method applies to samples characterized by increasing complexity, and its simplicity will contribute to its widespread use in plant and microalgae photoacclimation studies.

## Graphical abstract

**Figure:**
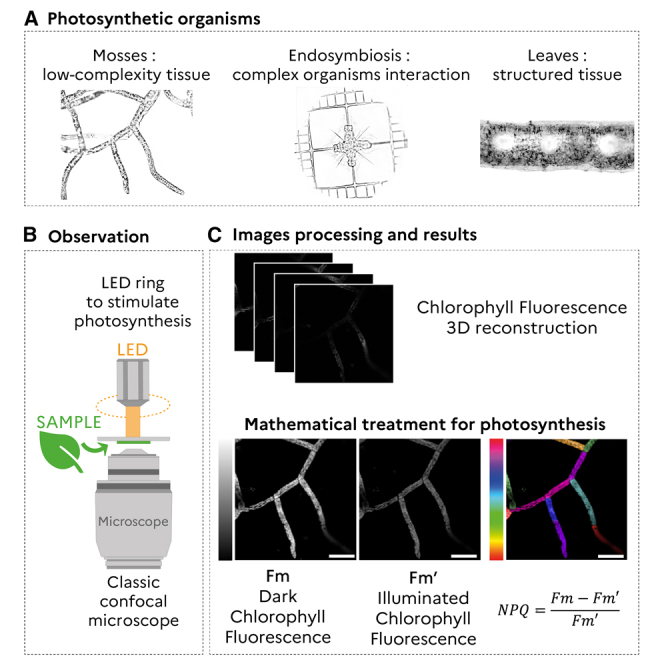

## Teaser

Storti et al. develop a method to study photosynthesis at single-cell resolution using confocal microscopy. They use this approach to examine photosynthesis specialization in different tissues and organisms and to understand the relationship between light penetration and photoprotection.

## Introduction

Photosynthesis is a major bioenergetic process in the biosphere. It feeds most of the food chains on Earth and is responsible for substantial sequestration of CO_2_ via the biological pump. The efficiency and regulation of this process is usually assessed *in vivo* by measuring chlorophyll (Chl) fluorescence,^1^ i.e., the fraction of absorbed light that is re-emitted in the near-infrared region of the spectrum. Chl fluorescence can be analyzed at different scales: from the cellular level using microscopes to organs/organisms using infrared cameras or even at larger scales (ecosystems/planetary) using satellites.^2^^,^^3^^,^^4^ Those approaches all suffer from a similar limitation, namely the detection of images in two dimensions only. However, significant changes are expected within the 3D volume of phototrophs, because the color and intensity of light vary according to its penetration into the absorbing/diffusing layers of photosynthetic cells.^5^ So far, theoretical approaches have been used to extrapolate data obtained from a surface (leaf, canopy, or ocean) to a volume, by modeling light penetration.^6^ In a few cases, the responses of a photosynthetic tissue (e.g., a leaf) to different colors of light (blue, green, and red) were compared to highlight the effect of different light penetrations on photosynthesis.^7^^,^^8^^,^^9^ Both approaches have limitations as they do not rely on direct assessment of photosynthesis inside an intact photosynthetic tissue/organ. Moreover, monitoring fluorescence changes in two dimensions does not allow detecting fluorescence of a single plastid, because these organelles move inside cells.^10^^,^^11^^,^^12^ To overcome this difficulty, we have explored the potential of a Chl fluorescence imaging approach that combines the spatial resolution of a confocal microscope with the reliability of the saturation pulse method.^13^ The latter approach has been particularly successful in assessing relevant photosynthetic parameters (the quantum yield of photosystem [PS] II in the dark [Fv/Fm] and in the light [ΦPSII]) and the thermal dissipation of excess excitation energy (NPQ) to study CO_2_ assimilation capacity,^14^ plant acclimation to the environment, and stress responses.^15^ We show that the 3D saturating pulse confocal setup provides unique physiological information concerning photoprotection in biological samples characterized by increasing complexity: (1) heterogeneous responses of single chloroplast/cell in mosses, which contain multicellular and partially differentiated tissues, (2) complex relationships in photosymbiosis between eukaryotic host cells and symbiotic microalgae at different developmental stages, (3) the link between leaf architecture and photoprotection in vascular plants.

## Results

### Combining a saturating pulse method with a confocal microscope

**Figure 1:**
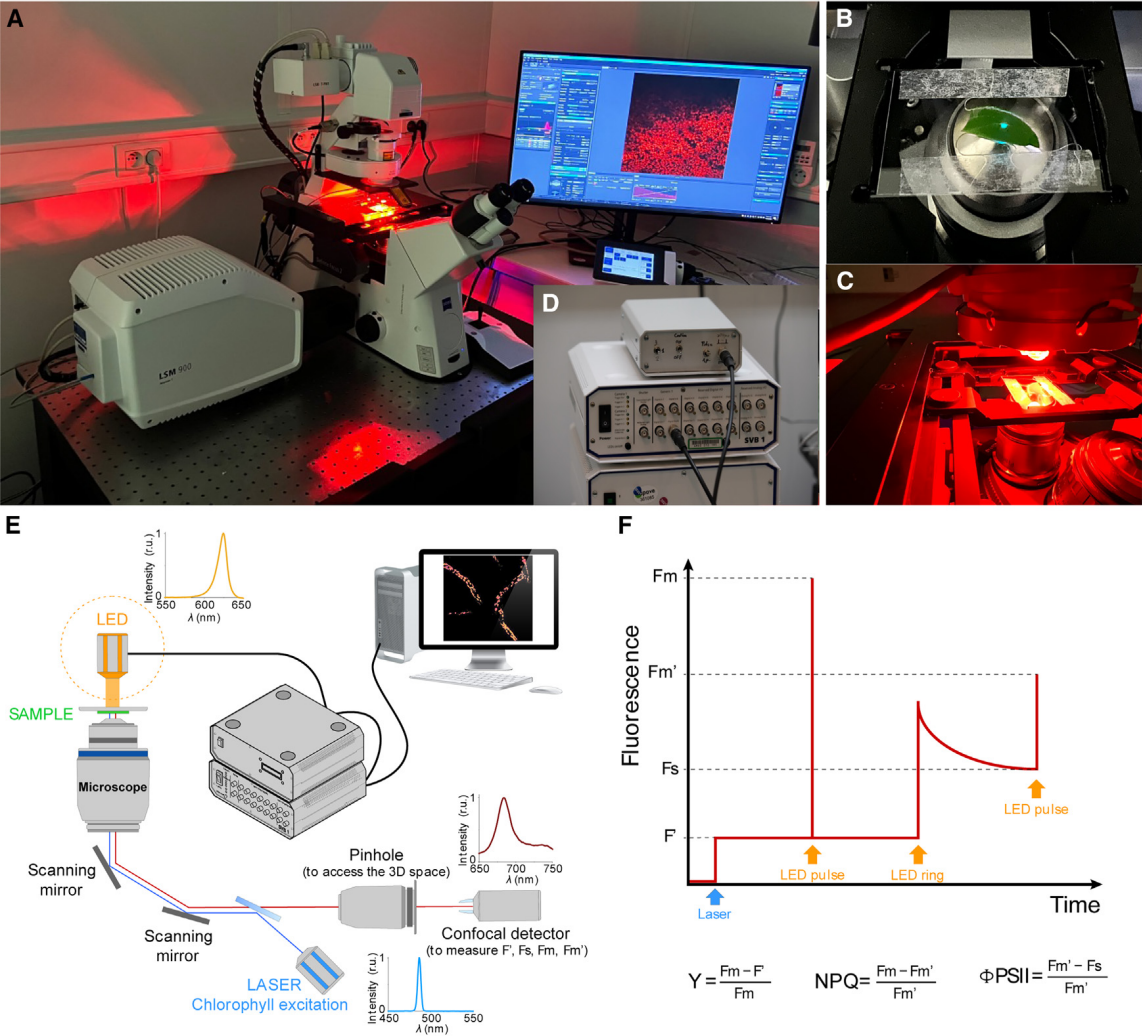
3D-Pulse fluorimeter imaging setup (A–D) Global view of the setup. (B) Close-up on the sample (here a piece of leaf) with the confocal laser on showing the small portion of the sample illuminated. (C). Close-up on the same sample with the actinic light on, showing an area of illumination larger than the sample. (D) Close-up of the control box and connections to the confocal system. (E) Scheme of the customized confocal microscope includes an orange LED array controlled by the Zen software via the SVB1 module plus a homemade controller to deliver actinic light and saturating pulses. (F) Switching on and off the light pulses via the “experimental design” routine (and manual switching on and off the actinic light) allows evaluating relevant photosynthetic parameters.

To develop a 3D saturating pulse confocal (hereafter 3D-Pulse fluorimeter, Figure 1A), we equipped a Zeiss LSM900 inverted confocal microscope (for alternative systems, see Table 1) with an additional red light LED source (λ = 630 nm, full width-half maximum 18 nm) placed in front of the microscope objective (Figures 1B–1E). The LEDs deliver, on the entire sample (Figure 1C), short and intense pulses (2,000 μmol photons m^−2^ s^−1^) to saturate PSII and thus achieve maximum Chl fluorescence emission, Fm.^14^^,^^16^ The LEDs also provide continuous actinic light of adjustable intensity (actinic light), to achieve steady-state (Fs) fluorescence (Figure 1F). Both the pulses and the continuous light delivered by the LED array are operated by a home-built control box (Figures 1D and S1). The blue laser (λ = 488 nm) of the confocal is used as the “measuring light” in the saturation pulse method^13^ to image Chl fluorescence (Figure 1E), leading to a parameter hereafter called Fʹ (Figure 1F). This parameter is close to the F0 parameter used in the saturating pulse method.^13^ Both the blue laser, the acquisition (on a predefined z stack) and the LED control box are controlled by the confocal software Zen (version 3.0), through the “experiment designer” routine. We used the Fiji software^17^ to analyze the datasets and treated the images as follows: 3D time series acquisitions (xyzt) were converted to 2D images (xyt) in a way that preserves original fluorescence values (“sum slices” function). The latter can be calculated for every time point measuring the “mean gray value” of regions of interest (ROIs) (chloroplasts, cells, and tissues) and subtracting the background fluorescence (i.e., the signal measured in an empty ROI located near the measurement region). Indeed, background fluorescence is also affected by external light source (Figure S2). When needed, image segmentation was done with the 3D Slicer software^18^ to generate 3D models that gave information about the plastids volume. We calculated photosynthetic parameters from fluorescence values with the Origin software (Microcal).

**Table 1:** Equipment needed to adapt our method on different confocal systems

Brand (by alphabetic order)	Confocal system	Electronic interface	Software module
Leica	STELLARIS series	trigger box 158004760 (ref. Leica)	LAS X live data mode 158203201 (ref. Leica)
Nikon	A1, AX series	Ti2 controller (Digital I/O)	NIS-Elements advanced interpreter (MQS42510)
Olympus	FLUOVIEW series	FV30-analog	Fluoview 3000
Zeiss∗	LSM series∗	SVB1 module∗	“experimental design” routine of the ZEN software (v.3.0)∗

Asterisks (∗) indicate the systems used in this study.

**Figure 2:**
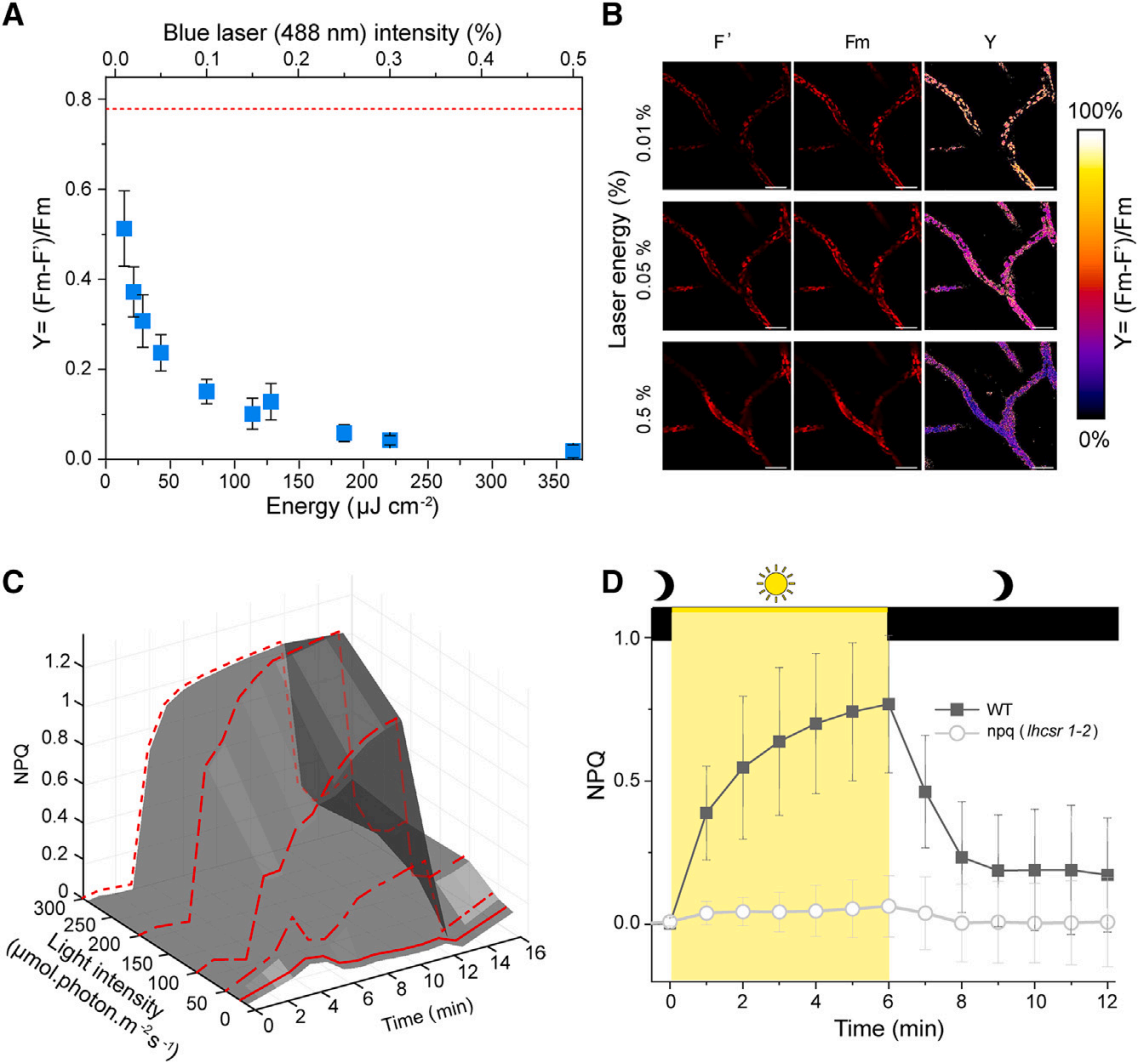
Validation of the 3D-Pulse fluorimeter (A) The apparent quantum yield of photosystem II (Fm-F′)/Fm is lower than values measured with a standard Chl fluorescence imaging camera (red dashed line), and decreases as a function of the energy of the confocal laser power (blue squares, n = 7 cells average ± SD). (B) Representative Chl fluorescence images (red) used to calculate Y (artificial color). Scale bar, 50 μm. (C) NPQ changes as a function of the light intensity (25, 50, 100, 200, and 500 μmol photons m^−2^ s^−1^). Representative traces of an experiment repeated 5 times with similar results. (D) NPQ features in a WT (solid symbols, average of 108 cells ± SD) and mutant (open symbols, average of 63 cells ± SD) with downregulated NPQ capacity. Dark box, actinic light off; yellow box, actinic light on (500 μmol photons m^−2^ s^−1^).

We first validated the 3D-Pulse fluorimeter on a photosynthetic organism having a relatively simple structure, the juvenile gametophyte (protonema) of the moss *Physcomitrium patens*. We noticed that the maximum photosynthetic capacity, here indicated by the PSII-related parameter Y = (Fm – F′)/Fm, decreased when the laser intensity increased. The Y decrease reflects the actinic effect of blue laser itself, which increases the F′ parameter. However, the lack of fluorescence quenching during exposure to multiple (four) laser pulses before the saturating pulse (Figure S2B) demonstrates that each measurement does not influence the subsequent one. At 0.3% laser intensity (i.e., 220 μJ cm^−2^, Figures 2A and 2B), the F′, obtained in the presence of the blue laser illumination alone, reached the same level as Fm, which is the fluorescence intensity obtained by concomitant illumination with the blue laser and the saturating red pulse. This finding suggests that the blue laser alone, which is localized over a very small area, saturates photosynthesis (inducing Fm) even at relatively low intensities. We exploited this possibility to measure Fm and Fm’ without the saturating pulse protocol, and therefore to calculate NPQ, which is readily estimated from these two parameters (Figure 1F). On the other hand, the relative PSII yield can be evaluated using a sub-saturating laser intensities (Figure 2B).

The 3D-Pulse fluorimeter was fast enough to measure the kinetics of NPQ onset (when the actinic light was switched on) and its relaxation in the dark (Figure 2C; Video S1). We could also detect changes in NPQ amplitude as a function of actinic light intensity, as well as a transient NPQ during exposure of the dark-adapted protonema filaments to low light intensity (Figure 2C, dash and dotted line). This transient NPQ reflects the link between activation of CO_2_ assimilation and photoprotective responses: at the beginning of illumination, when CO_2_ assimilation is largely inactive, part of the absorbed light is dissipated. Conversely, most of the absorbed photons are drained to CO_2_ assimilation in steady state (when the Calvin-Benson-Bassham cycle is fully active) and thus NPQ disappears.^19^ Finally, we could easily differentiate NPQ features of a WT and a mutant strain with reduced NPQ capacity (due to knocking out of the NPQ effector proteins LHCSR1 and LHCSR2) (Figures 2D, S3A, and S3B; Video S2).^20^ We noticed that NPQ was lower in the 3D-Pulse fluorimeter than in a conventional 2D imaging fluorimeter equipped with similar light sources and intensities (Figure S3A). While this difference could be caused by the lower amount of actinic light delivered inside the tissue (where the 3D-Pulse fluorimeter measures), the kinetic features that we observed (Figures 2C and 2D) are consistent with the occurrence of a genuine quenching process (NPQ)^21^ in all the investigated samples.

Video S1. Time course of fluorescence changes in *P. patens*, related to Figure 2 Four time-course Z-projections (15–20 slices) of *P. patens* protonema exposed for 6 min to 500 μmol photons m^−2^ s^−1^. Time series that acquired with 1 min delay are reproduced at 1 frame per second (fps). The sum of raw fluorescence values for the different stacks is represented in an artificial color scale bar on the top.

Video S2. Time course of fluorescence changes in *P. patens* NPQ mutant, related to Figure 2 Four time-course Z-projections (15–20 slices) of *P. patens lhcsr1-2* KO protonema exposed for 6 min to 500 μmol photons m^−2^ s^−1^. Time series that acquired with 1 min delay are reproduced at 1 frame per second (fps). The sum of raw fluorescence values for the different stacks is represented in an artificial color scale bar on the top.

### Photoprotective responses in non-vascular plants

**Figure 3:**
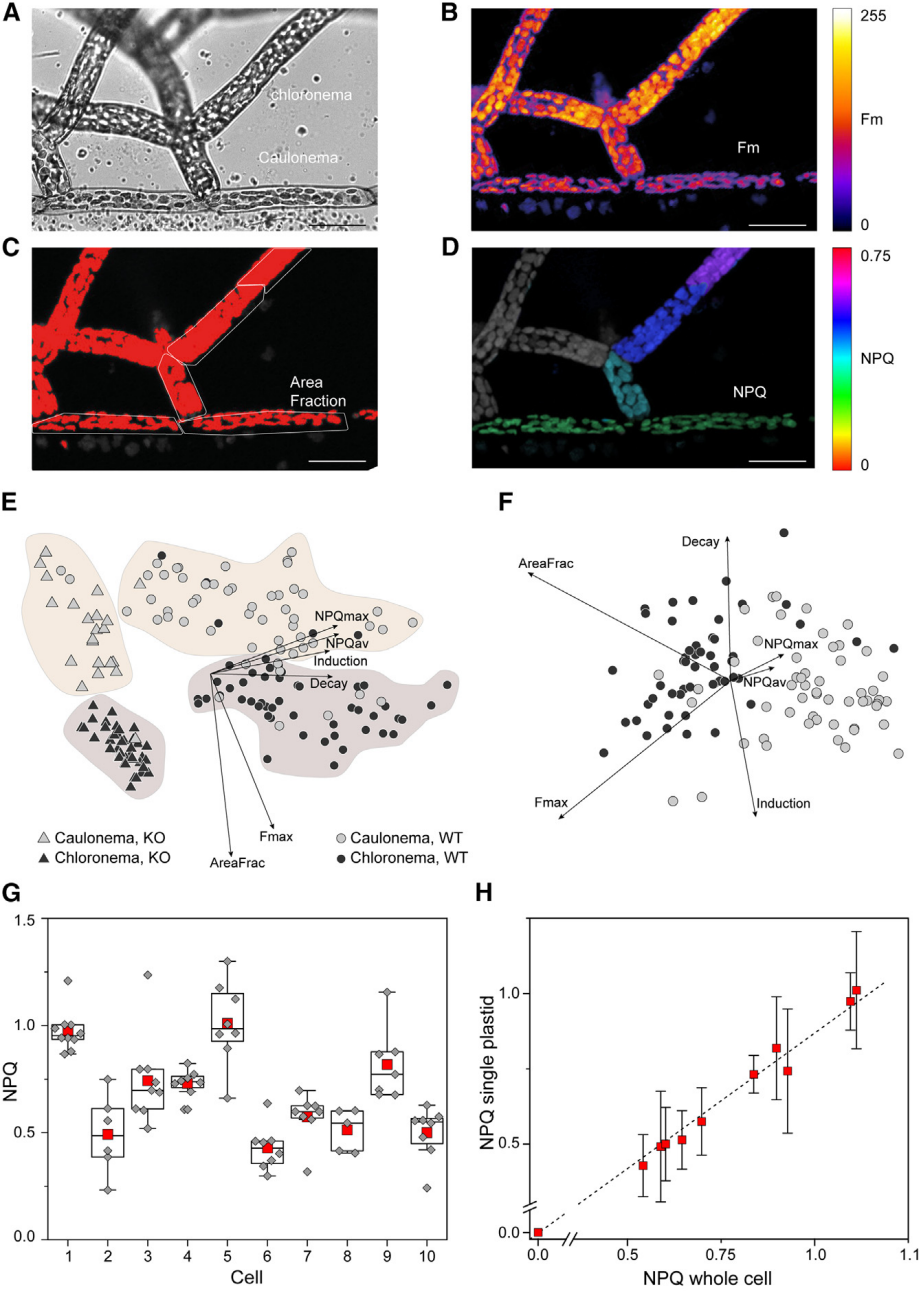
NPQ features in growing tissues of the moss *Physcomitrium patens* (A) Bright-field image. (B) Artificial color image of chlorophyll fluorescence. (C) Fraction of the cells occupied by plastids (red). (D) Artificial color image of NPQ in caulonema and chloronema cells of *P. patens* protonema. Scale bars, 50 μm. (E and F) Principal-component analysis of 175 cells (black circles, chloronema WT; black triangles, chloronema *lhcsr1-2* KO; gray circles, caulonema WT; gray triangles, caulonema *lhcsr1-2* KO). (E) First and second components and (F) second and third components for WT cells. The first two components represent roughly 88% of the variance, while the first three components represent more than 94% of the variance (Table S1). (G) NPQ is heterogeneous in *P. patens* cells. Red squares, mean of NPQ values of all plastids inside the same cell (gray diamonds; boxes, P25 and P75; whiskers, outliers; black line, median. (H) Plastid vs. cell NPQ relationship reveals that plastids (average of 5–10 ± SD) behave homogeneously inside a given cell.

We explored the possibilities offered by the 3D-Pulse fluorimeter to study cellular and subcellular NPQ responses in the two cell types that constitute the protonema of *P. patens*: the caulonema and the chloronema. The former has longitudinally elongated cells involved in propagation and nutrient acquisition, the latter has chloroplast-rich cells, usually considered as the photosynthetic part of the moss protonema (Figure 3A).^22^ As plastids move inside the cell,^10^^,^^23^ they tend to leave the field of observation in a conventional microscope during the relatively long time required for NPQ development (Video S3). Instead, we could follow plastid responses within the entire volume of caulonema and chloronema cells with the 3D-Pulse setup (Video S1), visualize Chl fluorescence (Figure 3B), cell fraction occupancy (Figure 3C), and quantify NPQ capacity (Figure 3D) of single cells and plastids. A principal-component analysis of 175 mutant and WT cells allowed to distinguish four classes (Figure 3E; Tables S1–S3): the WT (circles) and the NPQ mutant *lhcsr1-2* (triangles) were separated based on their NPQ capacity, while the two cell types (black, chloronema; gray, caulonema) could be differentiated because of their plastid cell density. A more refined analysis (WT, Figure 3F, see also Figure S3C for the *lhcsr1-2* mutant) revealed subtle heterogeneity in NPQ responses in both caulonema and chloronema, which we could interpret based on 3D imaging. We identified heterogeneous NPQ responses at the cell level (Figure 3G), which account for most of the above-mentioned heterogeneity. Conversely, single plastids (Figure S3D) within a given cell behave homogeneously (Figure 3H): cells with high photoprotective responses contain plastids with high NPQ capacity, while cells with low photoprotection have plastids with low NPQ. Such variability in NPQ responses at the cell level probably reflects the different physiological state of cells that continuously regenerate during protonema development. While it is relatively easy to distinguish caulonema from chloronema in the complex matrix of the protonema, it is difficult to attribute more specific cellular characteristics (such as their age), which certainly have an impact on photosynthetic behavior.

Video S3. Plastids movements inside a *P. patens* filament, related to Figure 3 Overlapped bright-field (gray) and chlorophyll fluorescence (red) images acquired with an upright Leica DM6 B (HC PL FLUOTAR 20×/0.50 objective, excitation filter 450 nm) optical microscope. Image size = 342.58 × 256.89 μm. Time series acquired with a delay of 30 s between different images and reproduced at 2 frames per second.

### Probing heterogeneous photosynthetic activity inside a complex, photosymbiotic organism

**Figure 4:**
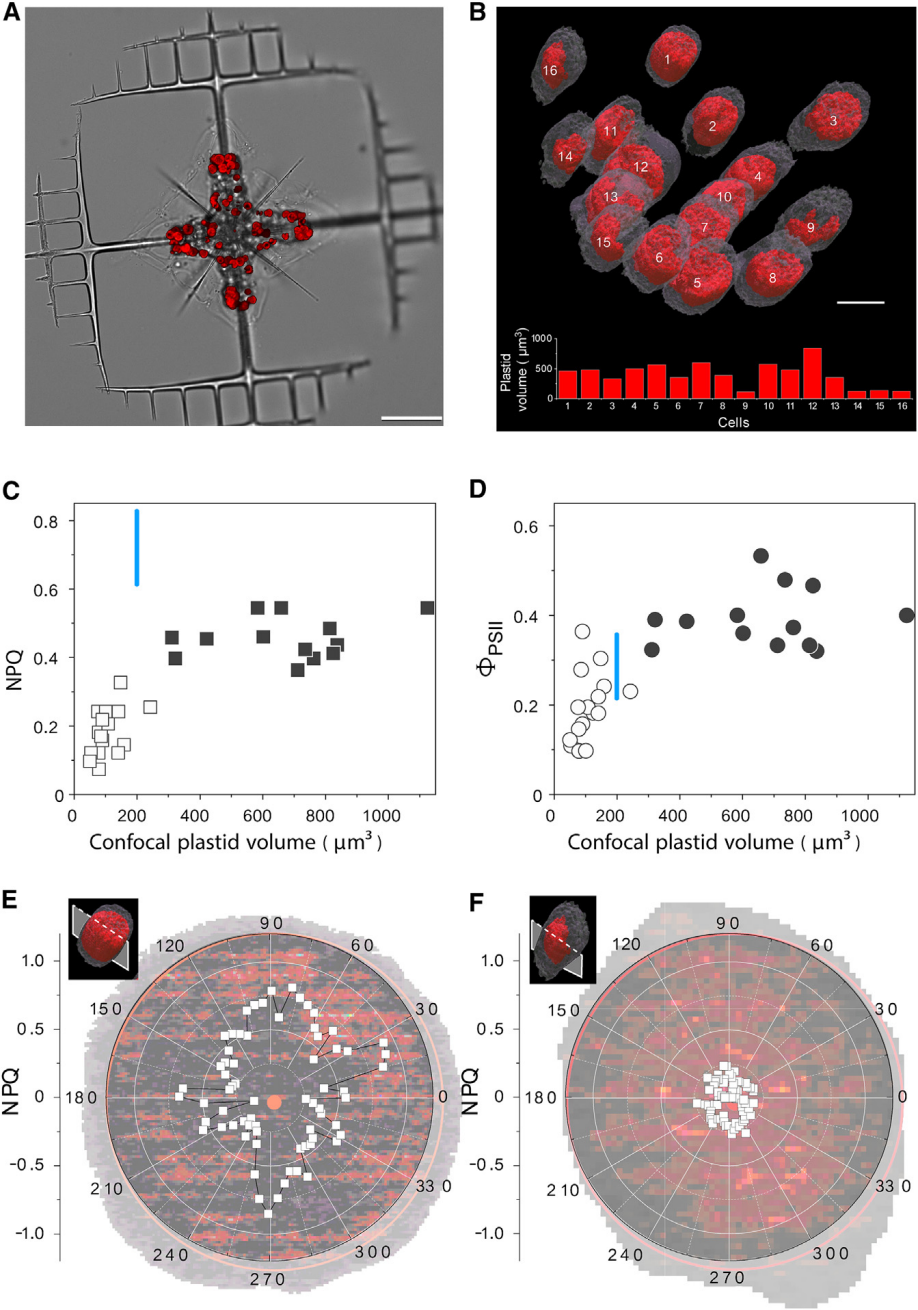
Photosynthetic features in a planktonic photosymbiosis (A) Bright-field (gray) and Chl fluorescence (red) images overlaid from a host acantharian cell harboring symbiotic microalgae (the haptophyte *Phaeocystis*). Scale bar, 50 μm. (B) 3D reconstruction of plastid chlorophyll fluorescence inside 16 microalgae (top) reveals large differences in their plastid volumes (bottom). Scale bar, 10 μm. (C and D) Single-cell analysis reveals the existence of two symbiont populations having different photoprotective responses (NPQ) and photosynthesis (ΦPSII); in white the small symbiotic cells, in black the large symbiotic cells. Blue bars, NPQ and ΦPSII in free living *P. cordata* cells (n = 15 ± SD). (E and F) Radar plot of the NPQ (white squares) in large (E) and small (F) symbiotic microalgae. NPQ was calculated from images acquired in the dark and after exposure to actinic light (500 μmol photons m^−2^ s^−1^) for 15 min. Representative traces of an experiment performed on eight cells (E) and five cells (F), respectively (Figure S4).

We further investigated the ability of the 3D-Pulse fluorimeter to link NPQ responses to different physiological states focusing on photosymbiosis, a common lifestyle in oceanic plankton between symbiotic microalgae and unicellular eukaryotic hosts.^24^^,^^25^^,^^26^^,^^27^^,^^28^ Inside hosts (acantharians), symbiotic microalgae (the haptophyte *Phaeocystis cordata*, Figure 4A) undergo progressive morphological and metabolic changes (i.e., multiplications of plastids). Therefore, a single host cell contains a mix of newly engulfed/small symbionts with two plastids, and of larger (presumably older) ones, with up to 60 plastids as revealed by focused ion beam scanning electron microscopy imaging.^29^^,^^30^ Using confocal microscopy, we confirmed the algal heterogeneity in terms of cell volume occupied by plastids (Figure 4B, histogram).^30^ Moreover, we were also able to extract quantitative photosynthetic characteristics of individual microalgae inside the host upon segmentation and 3D reconstruction of their Chl fluorescence emission (Figure 4B).

Relating plastid volume heterogeneity to single-cell NPQ responses revealed the existence of two symbiont populations: small algae (open symbols) exhibited lower NPQ (Figure 4C) and photosynthetic activity (assessed by the ΦPSII parameter, Figure 4D) than free-living cells in culture measured with the same 3D-Pulse fluorimeter (Figures 4C and 4D, blue bars). Conversely, larger symbionts (solid symbols) had a different trend: their NPQ was always lower, while ΦPSII was higher than in free-living cells. We interpret the lower NPQ but higher ΦPSII of larger symbionts as a signature of enhanced photosynthetic performance upon transformation of the alga inside the host, as reported previously.^29^ The physiological activity of the small symbiont population has not been reported so far in photosymbiosis, likely because it represents a relatively small fraction of the symbiotic cells, difficult to observe with conventional Chl fluorescence imaging setups. Their photosynthetic features (concomitant decrease of photosynthesis and NPQ) are reminiscent of the ones observed in the diatom *Phaodactylum tricornutum*^31^ and the green alga *Chlamydomonas reinhardtii*^32^ upon mineral nutrient (Fe) limitation. We propose that this particular population comprises microalgae in the process of adapting to the trophic environment provided by the host (see discussion).

We investigated their NPQ features at the subcellular level. However, it was difficult to track single *Phaeocystis* plastids separately because these organelles are around 10 times smaller than the ones found, e.g., in plants and *P. patens*.^33^^,^^34^ Moreover, they are very densely packed^30^ and thus cannot be easily separated based on confocal images. Thus, we developed an alternative data analysis approach: we first selected a 2D slice from the plastid 3D volume. Chl fluorescence values were calculated along the radius of a circular surface, approximating cell area. This was done for acquisitions of dark-adapted samples and for cells exposed to 10 min of illumination to evaluate the Fm, Fm’, and NPQ ((Fm – Fmʹ)/Fmʹ) parameter. We repeated this calculation on different radii (separated by 5°) to infer homogeneous/heterogeneous NPQ responses inside the cells. Thanks to this approach, we could highlight heterogeneous NPQ values in both large (Figure 4E) and small (Figure 4F) symbionts (see also Figure S4), only using a single NPQ measurement. This result suggests that plastids of photosymbiotic cells develop NPQ responses in a rather heterogeneous manner when compared, e.g., to mosses.

### Vascular plant NPQ is regulated by light channeling throughout anatomically diverse leaf architectures

**Figure 5:**
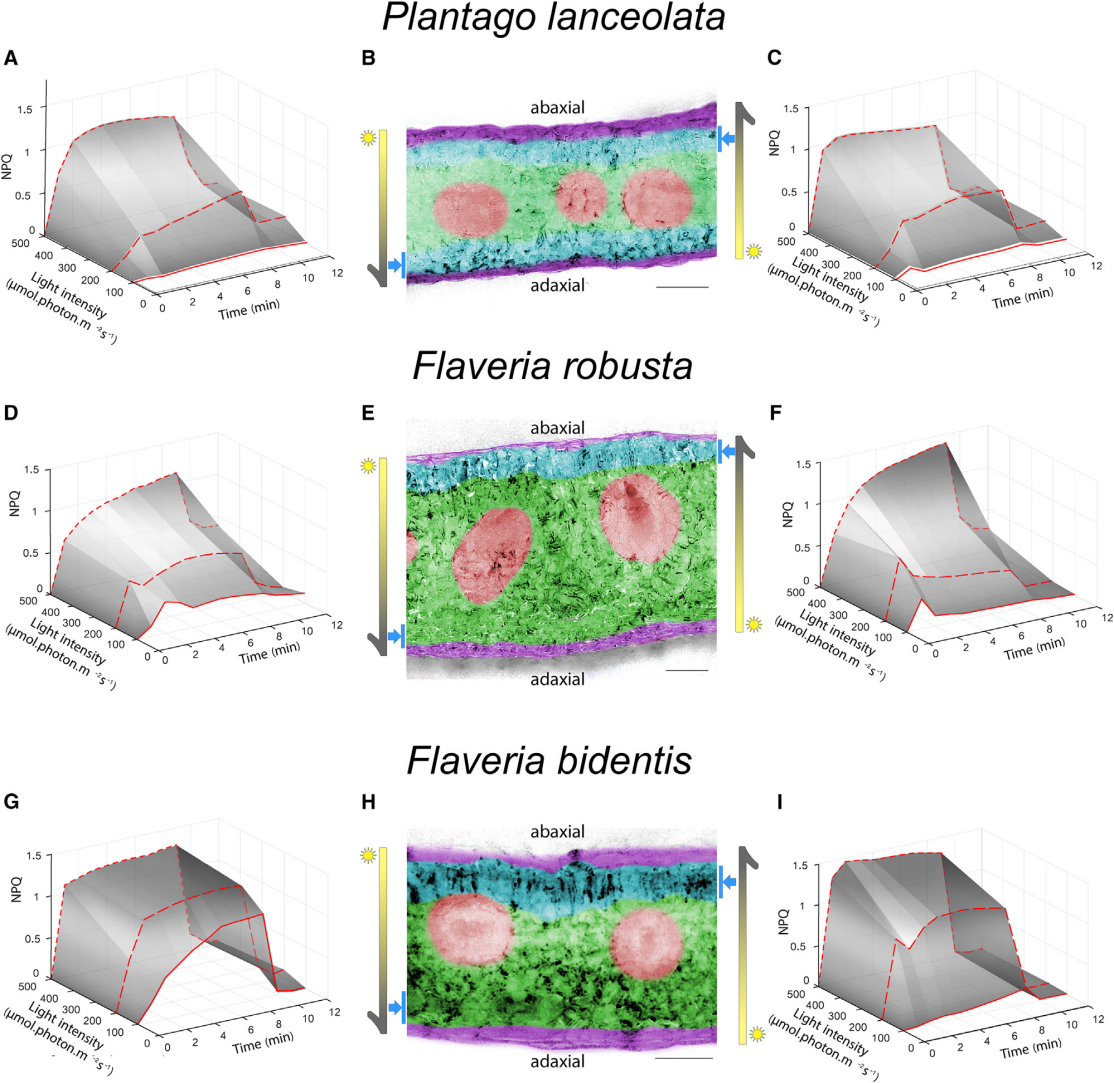
NPQ features are modulated by leaves’ architectures (A–I) NPQ measured upon illumination in the adaxial to abaxial side (top to bottom, A, D, and G) and abaxial to adaxial (bottom to top, C, F, and I) for three leaves with different anatomies. Representative picture of an experiment repeated 3–7 times with similar results. (B, E, and H) Artificial colors representation of the different leaf tissues: purple, epidermis; blue, palisadic parenchyma; green, spongy parenchyma; red, vascular tissue. Vertical bars with blue arrows represent the region imaged in NPQ experiments. Scale bars, 100 μm (gray).

Finally, we exploited the 3D-Pulse fluorimeter to investigate fluorescence responses in highly complex photosynthetic architectures: vascular plant leaves. These highly efficient machineries are composed of millions of cells, receiving variable light intensity depending on their position inside the organ. Cells on the surface receive more photons than cells inside the leaf, resulting in a light gradient.^35^ The light gradient in turn leads to a different extent of saturation of photosynthesis, which we inferred via the amount of light in excess dissipated via NPQ. Thanks to high resolution of the 3D-Pulse fluorimeter, and the penetration of the blue laser, we could measure NPQ in mesophyll cells around 80 μm under the epidermis (vertical bars with blue arrows in Figure 5) of a leaf exposed to the red actinic light on the opposite side of the detection. In a monocotyledon (*Plantago lanceolata*), we recorded similar NPQ responses on the adaxial and abaxial sides (Figures 5A and 5C) consistent with the observation of a symmetrical leaf morphology (Figure 5B), and thus of similar light gradients in both directions (Figure S5).

Conversely, we observed heterogeneous NPQ responses in dicotyledons (*Flaveria robusta* and *Flaveria bidentis*), which harbor an asymmetric mesophyll organization (Figures 5E and 5H): on the adaxial face, the palisade parenchyma (blue) under the epidermis (purple), is made up of elongated photosynthetic cells arranged perpendicular to the leaf surface. On the opposite abaxial side, this layer is replaced by the lacunar parenchyma (green), which occupies a large part of the leaf surrounding the vascular tissues (red) and consists of more irregular cells and large intercellular spaces to promote gas circulation and storage.

In both Flaveria species, NPQ at non-saturating light intensities was higher when actinic light was provided from the adaxial side to abaxial side (top to bottom, Figures 5D and 5G) compared with actinic light provided from the abaxial to the adaxial side (bottom to top, Figures 5F and 5I). By combining our 3D-Pulse fluorimeter derived findings with a more “classic” approach (measuring light penetration gradients inside a leaf,^6^ Figure S5) we interpreted these data as follows: thanks to channeling through the parallel cell layers of the palisade mesophylls, light better penetrates the leaf in the adaxial to abaxial direction (Figure S5). Therefore, it saturates photosynthesis at a lower photon density and induces high photoprotective responses (NPQ) (Figure S6). Conversely, illumination in the abaxial to adaxial direction appears to favor light scattering from randomly oriented spongy cells. This phenomenon decreases photon penetration, lowers excess light, and therefore NPQ. Consistent with this conclusion, we observed that the dissymmetry of the NPQ was exacerbated in *F. bidentis*, where the steepness of the light gradient is higher due to a reduced thickness of the leaf (Figure S5). In agreement with this notion, differences in NPQ were erased when the light was increased to 500 μmol photons m^−2^ s^−1^, an intensity that should over-saturate photosynthesis regardless of the direction of illumination. Hence, our method allowed characterizing *in situ*, without the use of models or assumptions, the effect of differential light penetration on photosynthesis.

## Discussion

In this work, we show that imaging Chl a fluorescence with a 3D saturating pulse confocal setup is well suited to image photosynthetic responses in three dimensions. Previous attempts to use a confocal microscope to measure photosynthesis^36^^,^^37^ suffer from the limitations of using the confocal laser as the “measuring light,” the “actinic light,” and the “saturating pulse” of the saturating pulse approach. This choice implies that two different lights cannot be provided at the same time (unlike in the saturating pulse approach), thus hampering the accuracy in the determination of the photosynthetic parameters and the number of possible applications (e.g., discriminating the stomata from epidermis in plant leaves, assessing single plastid fluorescence transients).^36^^,^^37^ Conversely, our setup combines the sensitivity and flexibility of the saturation pulse method with the 3D spatial resolution of confocal microscopy to investigate photosynthesis *in vivo* at the organ, cell, and subcellular levels. Previous work concluded that chloronema cells fix carbon within the protonema, while caulonema cells are mostly involved in nutrient propagation and acquisition.^38^^,^^39^^,^^40^ Our analyses indicate that the photosynthetic differences between these two cell types cannot be attributed to the intrinsic properties of PSII since photosynthetic parameters derived from PSII analyses (NPQmax, NPQav, decay, and induction parameters) are similar. Instead, we confirm that the net amount of the photosynthetic machinery as shown by the cell fraction occupied by plastids (Figure 3F, AreaFrac parameter) and the total Chl fluorescence emission (Figure 3F, Fmax parameter) is different for the two tissues. These differences, together with previously reported metabolic and transcriptomic ones in these two types of cells,^39^^,^^40^ likely explain the difference between caulonema and chloronema in the global process of carbon fixation.

In acantharians, we identified a particular algal subpopulation characterized by very low photosynthetic performance and photoprotection capacity. In general, these two parameters show complementary responses: low photosynthesis results in high dissipation of excess light in the form of NPQ, whereas high photosynthesis leads to low dissipation of excess light. The latter behavior is indeed observed in the large cells (Figures 4C and 4D), which probably represent algae that have established fully metabolic connections with the host.^30^ Conversely, the population with low photosynthetic performance is made up of small cells, probably still adapting to the host trophic environment.^30^ Indeed, a concomitant decrease in photosynthesis and photoprotection responses (NPQ) has only been reported in microalgae exposed to mineral nutrient (Fe) limitation.^31^^,^^32^ It is tempting to draw a parallel between this population, which likely represents a transient stage in the establishment of photosymbiosis, and the hypothetical early stage of endosymbiosis.^41^^,^^42^^,^^43^

Our analysis of leaf photosynthesis supports previous findings that light is differentially channeled through different leaf tissues.^6^ However, we go one step further, showing that moderate light intensity, which is on average received by most leaves in a tree due to mutual shading, can saturate photosynthesis over the entire leaf section in asymmetric leaves (Figures 5E and 5F). This is, however, only true when photons are captured on the adaxial side, where the palisade parenchyma channels them toward the opposite side of the leaf (Figures 5D and 5G), the spongy parenchyma. Overall, these results confirm the notion that leaf photosynthesis is largely governed by its anatomical features^44^ and further extends it to the cellular level.

### Limitations of the study

Our 3D imaging approach has clear advantages over conventional PAM fluorometers and single-cell pulse-probe microscopes, since it allows (1) disentangling heterogeneous cellular responses within a tissue (Figure 3), (2) tracking photosynthetic changes during cell development (Figure 4), (3) assessing the relationship between leaf anatomy (asymmetric or symmetric) and photoprotection (Figure 5). Its 3D resolution allows following the movements of plastids during prolonged exposure to light (Videos S1 and S2) to monitor their physiological responses.

However, there is a main difficulty associated with this approach. The choice of the laser light intensity is essential to ensure correct measurements of the photosynthetic parameters. In our case, the maximum photosynthetic capacity (the “Y” parameter in Figure 1F) decreases as a function of laser intensity (Figure 2A), as PSII becomes inactive (light saturated). The PSII is completely saturated (i.e., the Y parameter goes to 0) at 0.3% of the maximum power, indicating that the laser is too intense. In this case, this difficulty could be alleviated by placing neutral filters between the laser and the sample to decrease the laser power. Other organisms (or setups) may have different responses to light, so an experiment similar to that shown in Figure 2A is recommended before undertaking NPQ measurements.

## STAR★Methods

### Key resources table

**Table:** 

REAGENT or RESOURCE	SOURCE	IDENTIFIER
**Experimental models: Organisms/strains**		

Physcomitrium patens Gransden	Gerotto et al.^20^	N/A
Physcomitrium patens lhcsr1-2 KO	Gerotto et al.^20^	N/A
Flaveria robusta	HHU, Dusseldorf	N/A
Flaveria bidentis	HHU, Dusseldorf	N/A
Plantago lanceolata	HHU, Dusseldorf	N/A
Phaeocystis cordata	Decelle et al.^29^	Roscoff Culture Collection RCC1383
Acantharia	Decelle et al.^25^ Villefranche-sur-Mer	N/A

**Software and algorithms**		

ZEISS ZEN 3.0 (blue edition)	Carl Zeiss Microscopy	https://www.zeiss.com/microscopy/fr/produits/logiciel/zeiss-zen-core.html
Fiji	Schindelin et al.^17^	https://ImageJ.net/software/fiji/downloads
OriginPro version 9.0	OriginLab Corporation, Northampton, MA, USA	https://www.originlab.com/
3D Slicer - Slicer 4	Fedorov et al.^45^	https://www.slicer.org/
R software	R Core Team	https://www.R-project.org/.
Custom Code	Clarisse Uwizeye. (2023). compute-NPQ (v.1.0.0)	https://doi.org/10.5281/zenodo.8155231

**Other**		

LED module	JBeamBio, La Rochelle, France	N/A

### Resource availability

#### Lead contact

Further information and requests for resources and reagents should be direct to and will be fullfilled by the lead contact, Dimitri Tolleter (dtolleter@gmail.com).

#### Materials availability

This study did not generate new unique reagents.

### Experimental model and subject details

#### Photosynthetic models used in this study

*Physcomitrium patens* Gransden wild-type (WT) strain and *lhcsr1-lhcsr2* KO (*lhcsr1-2*)^20^ were grown on PpNO3 (3 mM Ca(NO_3_)_2_, 1 mM MgSO_4_, 50 μM FeSO_4_, 200 μM KH_2_PO_4_ pH 7, traces elements)^46^ solidified media (0.8% Agar type A suitable for plant cell culture) overlaid with a cellophane filter. Moss tissue was propagated vegetatively by homogenization through a tissue blender and cultivated in axenic condition at 25°C, 40 μmol photons m^2^ s^−1^ continuous illumination. 10-days-old moss protonema was employed for confocal microscopy measurement.

*Non treated seeds of Flaveria robusta*, *Flaveria bidentis* and *Plantago lanceolata* has been sown on soil (Floradur B fin, Soufflet Vigne, Puteaux, France) and grown 3–4 weeks in controlled growth chambers in long day conditions (16 h light/8 h dark) at a PPF of 100 μmol photons m^−2^ s^−1^. Air temperature was 22°C during the day and 21.0 °C at night. Relative humidity was constant at 70% during the day and night. Young fully developed leaves were chosen for the experiments.

Symbiotic acantharians harboring intracellular microalgal cells (*Phaeocystis cordata*) were gently collected by towing a plankton net of 150 μm in mesh size with a large cod-end (1 L) for 1–2 min in surface waters (Mediterranean Sea, Villefranche-sur-Mer, France). After collection, individual cells were isolated under a binocular with a micropipette.^25^ Cells were rapidly transferred to natural seawater and maintained at 20°C and 100 μmol photons m^2^ s^−1^ controlled illumination. Samples were imaged within 24 h from sampling time. Cultures of the haptophyte *P. cordata* (the symbiont of Acantharia in the Mediterranean Sea algal,^29^ strain RCC1383 from the Roscoff Culture Collection) were maintained at 20°C in K5 culture medium at 100 μmol photons m^−2^ s^−1.^20°C.

### Method details

#### Sample preparation

*P. patens.* A small portion (∼5 mm diameter) of *P. patens* protonema was endorsed on a 20 × 20 mm coverslip and soaked in 100 μL of water in order to spread the filaments. The coverslip was fixed to the microscopy slide by using a double-side tape of 0.15 mm thickness and finally sealed using VALAP (1:1:1 Vaseline, Lanolin, Paraffin) to prevent water evaporation.

*P. cordata* and *Acantharians* were settled on a microscopy glass bottom dish (Ibidi, Germany) directly on the microscopy plate to avoid media perturbation.

*F. robusta*, *F. bidentis* and *P. lanceolata*. 5 × 3 mm sections (longer axis parallel to major leaf veins) were excised using a scalpel. Sections were enclosed between two 20 × 20 mm coverslips allowing to observe both sides of the leaves. A double layer of tape was used to create an enclosure to lodge the leaf section and prevent its crushing. Water was provided to the sample to prevent dehydration during NPQ measurements.

All samples were dark adapted for at least 20 min before introduction in the setup.

#### Confocal microscope setup

The Zeiss LSM 900 microscope was equipped with continuous light and pulses of strong actinic light, provided by a LED module located in front of the biological sample. The module contains 4 red LEDs (OSRAM, LA W5a.m., λ = 630 nm, Full Width-Half Maximum 18 nm), equipped with a lens to reduce their divergence. The 4 LEDs were oriented at 42°, to focus their light onto the middle of the sample holding slits. The LEDs deliver actinic light, the intensity of which (100 and 200 and 500 μmols photons m^−2^ s^−1^) is determined by a 3-position switch located on the front face of the control box. Electronic diagrams of the control box are shown Figure S1. Whenever needed, lower actinic radiation levels were obtained by placing a neutral filter (Kodak ND0.3) between the LED array and the sample. This output is connected to the SVB1 Zeiss module, which controls fluorescence acquisition via confocal microscope through the ‘experimental design’ routine of the ZEN software (version 3.0 Blue edition). The same routine also triggers the switching on of saturating pulses (2000 μmols photons m^−2^ s^−1^, duration 1.1 s) to measure of Fm and Fm’. Alternatively, saturating pulses can be switched on manually through a button located on the control box. Refer to Table 1 to install the system on another type of confocal microscope platform than the one described above.

Images were acquired with a 20x/0.8 M27 objective (Zeiss) in confocal mode setting with Airyscan 2 detector. This setup has been chosen for fast acquisition without loss of sensitivity, thanks to the use of a collimation optic. Excitation was provided by a blue laser (488 nm) and Chl fluorescence was collected between 650 and 700 nm. Searching for the object under the microscope was conducted under low light intensity conditions (5 μmol photons. m^−2^. s^−1^ in white light) to minimize any potential irreversible impact on photosynthesis. Similarly, the determination of z-stacks boundaries was performed at an extremely low intensity level (30 times lower than the one utilized during the experiment) to ensure the integrity of the experiment and prevent undesired effects. The initial z position was set as the first slide where the fluorescence of chloroplasts, derived from chlorophyll fluorescence, could be clearly distinguished from the background. The initial z position was set as the first slide where the fluorescence of chloroplasts, derived from chlorophyll fluorescence, could be clearly distinguished from the background.^21^^,^^47^ A maximum of 25 z-stacks (512 x 512 pixels, z step size 2 μm) were acquired at the fastest speed (pixel dwell time 1.03 μs, with an illumination of 633 ms per frame), to minimise light exposure and therefore reduce the risk of sample photoinhibition during measurements.

To estimate maximum photosynthetic capacity (Y), a series of 10 consecutive images was acquired (experimental time 6.33 s). A saturating pulse was provided by the external LED source after the fourth acquisition (Figure S2A).

In the case of NPQ measurement, appropriate Z-stacks (xyzt experiment) were selected to include entire cells and maintain plastids in the acquisition field. The blue laser intensity was set at the minimum value (0.3% - 220 μJ cm^−2^) at which PSII fluorescence reached Fm to avoid excess excitation. The delay for two consecutive acquisitions was set to 1 min to prevent photoinhibion due to laser exposure. 3 points were acquired for dark adapted samples, 6 during exposure to actinic red light (LED module) and 6 points again in the dark to follow NPQ relaxation (experimental time 15 min, Figures S2C–S2F). Stability of Fm measurement (and so NPQ) have been checked during the first 2 min of the experiment (in the dark) confirming the relaxed state of the NPQ.

To calculate the ΦPSII parameter, lower intensities of the actinic laser were chosen, in order not to reach Fm, but rather a steady state level Fs. Maximum fluorescence Fm was instead achieved when the laser and the saturating Orange LED were switched on simultaneously.

In Figures 2A and S3B, Chl fluorescence was instead measured with a conventional imaging setup (Speedzen, JBeamBio, France) described *e.g*., in Seydoux et al.^48^

### Quantification and statistical analysis

#### PSII yield and NPQ calculation

Experimental files were imported to Fiji,^17^ using the “sum slices” routine and we transform xyzt files into xyt to calculate a time course of fluorescence changes in case of 3D acquisitions. Regions Of Interest (ROIs) were drawn on the Z project image to select single chloroplasts, cells or whole tissues or non-fluorescent regions that were used to estimate the background level (Figure S2A). ‘Mean gray value’ of fluorescence was quantified for each single ROI and time. Background was subtracted to raw values for each time point. Numeric fluorescence values were imported to Origin software (Microcal, USA) to calculate PSII related parameters. In the case of PSII capacity (Y), F′ value is the mean value for dark adapted samples measured with non-saturating laser light. Fm is instead the maximum value of the fluorescence obtained during exposition to a saturating pulse (Figures S2A and S2B). For NPQ, Fm is the mean value for dark adapted plant exposed to saturating laser intensity; Fm’ is the maximum fluorescence value samples exposed to actinic light or during the dark relaxation time (Figure S2B). In the case of acantharians (Figure 4), Fs is the fluorescence measured in the presence of the non-saturating blue laser, while Fm is the fluorescence value achieved in the presence of the laser plus the saturating value of the orange LED.

#### 3D reconstruction and fluorescence integration

Image processing was done adapting a pipeline previously developed for 3D reconstruction based on electron microscopy stacks.^18^ Briefly, confocal images were pre-processed using a non-linear median filter (from Fiji) that preserves the edges. This is an essential prerequisite to calculate object volumes (see *e.g*., Figure 4B). We used 3DSlicer to perform semi-automatic segmentation.^45^ To calculate the volume of a reconstructed 3D model, we multiplied the number of voxels (volumetric picture elements) in the object by the size of the voxel: VI = (number of voxels in the object I) × (size of the voxel Vi)

To calculate the fluorescence of each object in a confocal image, images were segmented to obtain the location of a given ROI). Data were used to calculate the sum of the voxel values of a given ROI, and therefore the mean fluorescence value of a given plastid or cell. In the case of *Phaeocystis cordata* photosymbiotic cells, where single plastids could not be imaged because of their small size, fluorescence parameters were calculated on cell sections, which we approximated with a circle (red circle in Figures 4E and 4F). To assess possible heterogeneous responses, we scanned fluorescence values along the radius. We then interpolated the corresponding fluorescence intensity to calculate fluorescence parameters (Fm, Fm’), and therefore NPQ, as a function of the angle. At least ten sections (*i.e*., 10 μm) were scanned for every cells to assess reproducibility.

#### Principal components analysis (PCA)

We performed PCA considering six observed variables: NPQav, NPQmax, Decay, Induction, Fmax and Area Fraction. The variables used here were calculated in automatized way from NPQ data. NPQav is the mean NPQ when a cell is exposed to light while NPQmax is the maximum of NPQ reached during the light exposition. Decay and Induction are the relaxation and induction rate of NPQ evaluated from the slope of NPQ changes during the first 2 min of the light to dark and the dark to light transitions, respectively. Area Fraction is the area percentage occupied by the chloroplast inside a cell. Fmax is the “Mean gray value” of fluorescence of a given cell chloroplasts for dark adapted samples (Fm).

Variables were measured in 175 cells from *P. patens* chloronema and caulonema in the WT or the *lhcsr1-2* KO strains. The type of cells (mutant/WT or chloronema/caulonema) did not play a role in the determination of the component and that they are used after to characterise the possible biological role of the components. All data are normalized by subtraction of the mean and division by the standard deviation so that the singular values decomposition is done on the correlation matrix of the data (Table S1).

To represent the distribution of these normalized dimensional data for the 175 images, the direction (a 6-dimensional vector) giving the largest possible variance of the distribution was selected as the direction for the first principal component. Then, we selected the direction orthogonal to the previous one(s) giving the largest possible variance of the distribution as the direction for the second principal component. By repeating this procedure automatically, we identify vectors representing the scatter of the distribution from major ones to minor ones (Table S2). Based on singular values decomposition, PCA is a principal axis rotation of the original variables that preserves the variation in the data. Therefore, the total variance of the original variables is equal to the total variance of the principal components. The principal component coefficients correspond to the percentage of explained variance. Statistical analysis was done with the R software (http://www.R-project.org). The table of the original observed variables used to construct the six components (Table S3) provides the interpretation of the components.

## Data Availability

All data reported in this paper will be shared by the lead contact upon request. Original code is available at this address Clarisse Uwizeye. (2023). compute-NPQ (v.1.0.0). Zenodo. https://doi.org/10.5281/zenodo.8155231 Any additional information required to reanalyze the data reported in this paper is available from the lead contact upon request.

## Supplemental information

Document S1. Figures S1–S6 and Tables S1–S3

Document S2. Article plus supplemental information
