# Supplementary material for: Tailoring confocal microscopy for real-time analysis of photosynthesis at single-cell resolution
Source: Cell Rep Methods. 2023 Aug 28;3(9):100568. doi: 10.1016/j.crmeth.2023.100568 (PMC10545909; doi:10.1016/j.crmeth.2023.100568)
Supplement: Document S1. Figures S1–S6 and Tables S1–S3 [file mmc1.pdf]

Cell Reports Methods, Volume *3*

Supplemental information

Tailoring confocal microscopy for real-time analysis of photosynthesis at single-cell resolution

Mattia Storti, Haythem Hsine, Clarisse Uwizeye, Olivier Bastien, Daniel P. Yee, Fabien Chevalier, Johan Decelle, Cécile Giustini, Daniel Béal, Gilles Curien, Giovanni Finazzi, and Dimitri Tolleter

A

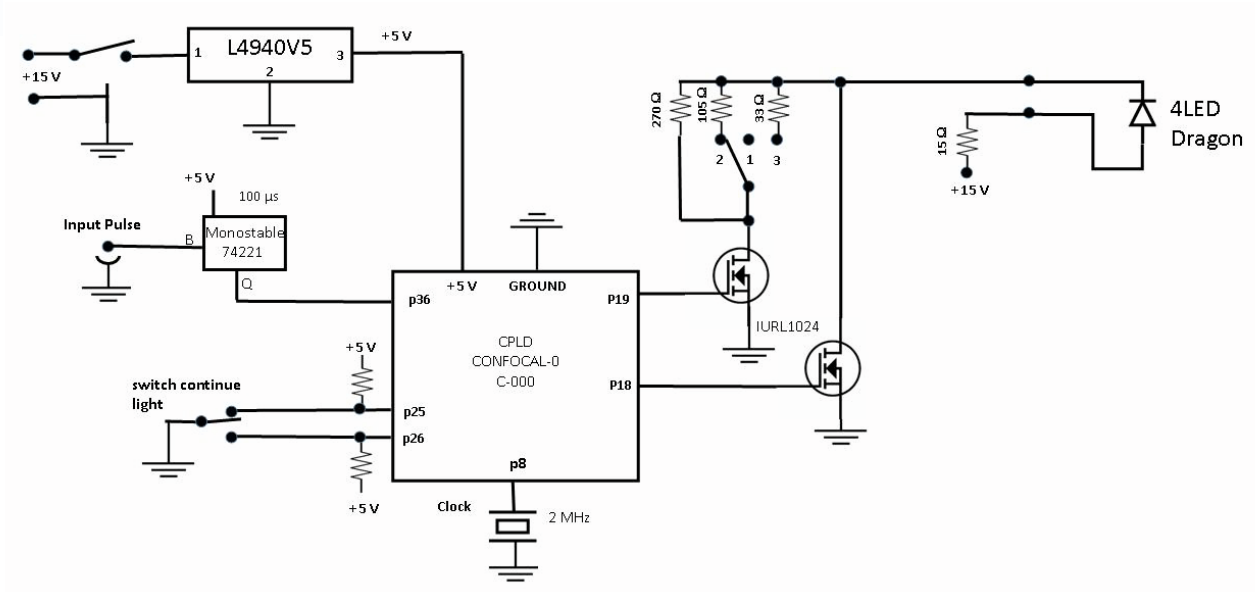

B

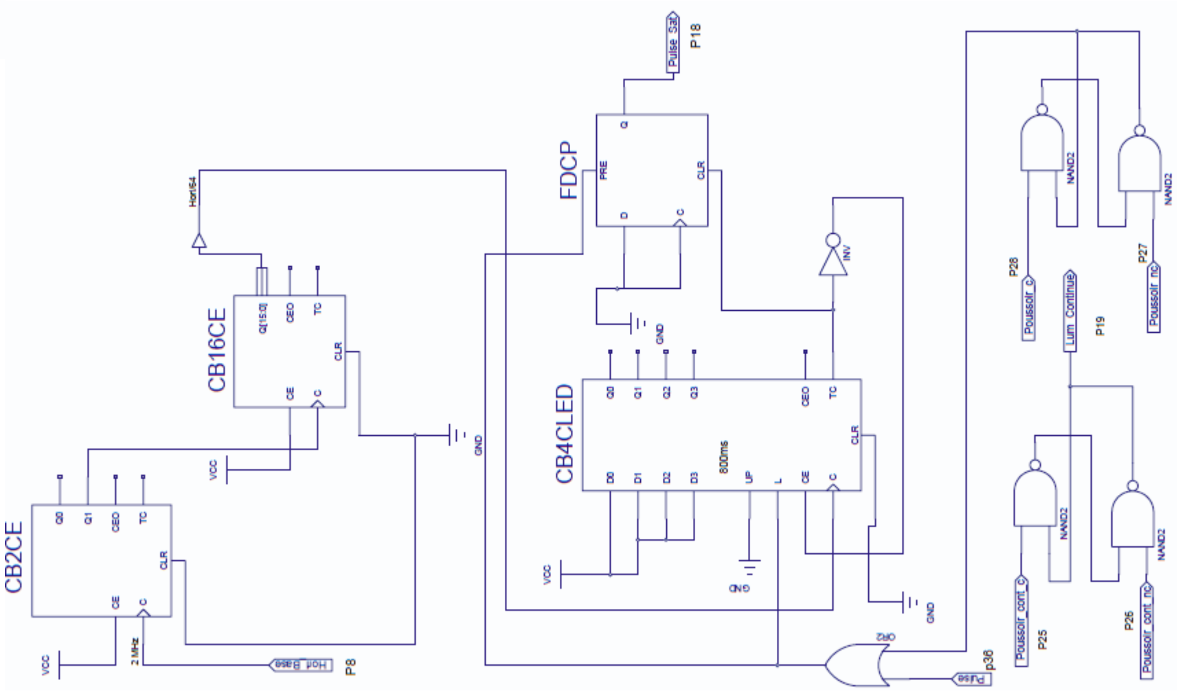

Figure S1 (related to Figure 1): Electronic scheme of the control box. (A), general scheme, **(B)**, details of the CPLD**.**

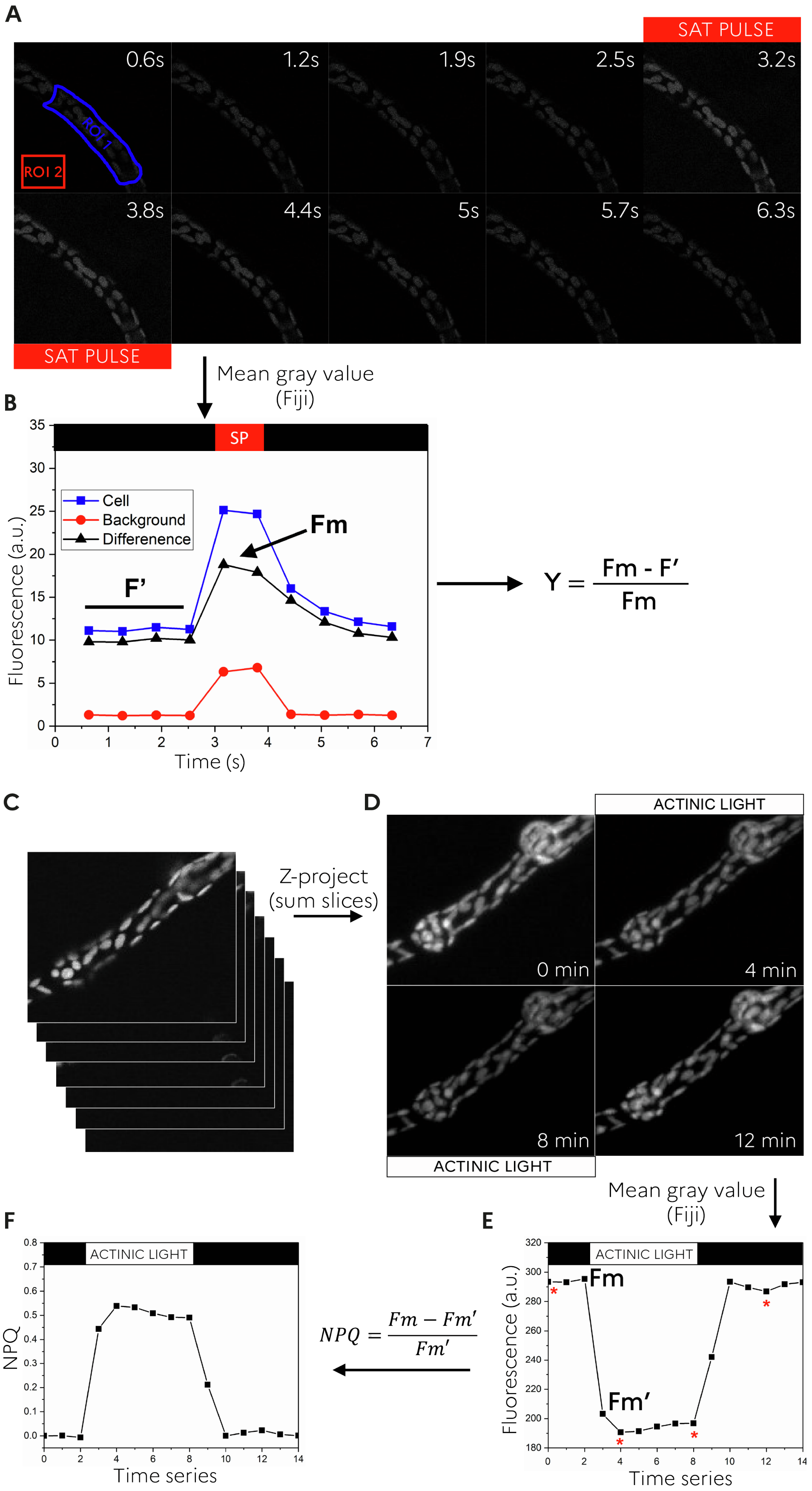

Figure S2 (related to Figure 2): Analysis of confocal images with Fiji. (A), To measure the ‘Y’ parameters (PSII yield) we calculated the “mean gray value” of ROIs (ROI 1, blue: *P. patens* cell; ROI 2, red background). **(B)**, Background fluorescence (red), which is altered by external light source (Saturating pulse, SP, red bar) was subtracted from the sample fluorescence (blue) value to obtain the real fluorescence value (difference, black). By plotting the results of this calculation for sequential acquisitions, we obtained a time series, which we used to calculate Y values. For NPQ experiments, xyzt files **(C)** were converted to xyt files **(D)** using the “sum slices” function of Fiji. Images were analysed as in (A) to obtain fluorescence **(E)**. Fm is the fluorescence value of the dark-adapted sample (top black bar) while Fm’ is the fluorescence value measured during exposition to actinic light (top white bar). Both values are needed to calculate NPQ **(F)**. Red asterisks in (E) represent the fluorescence values calculated from the images represented in panel D.

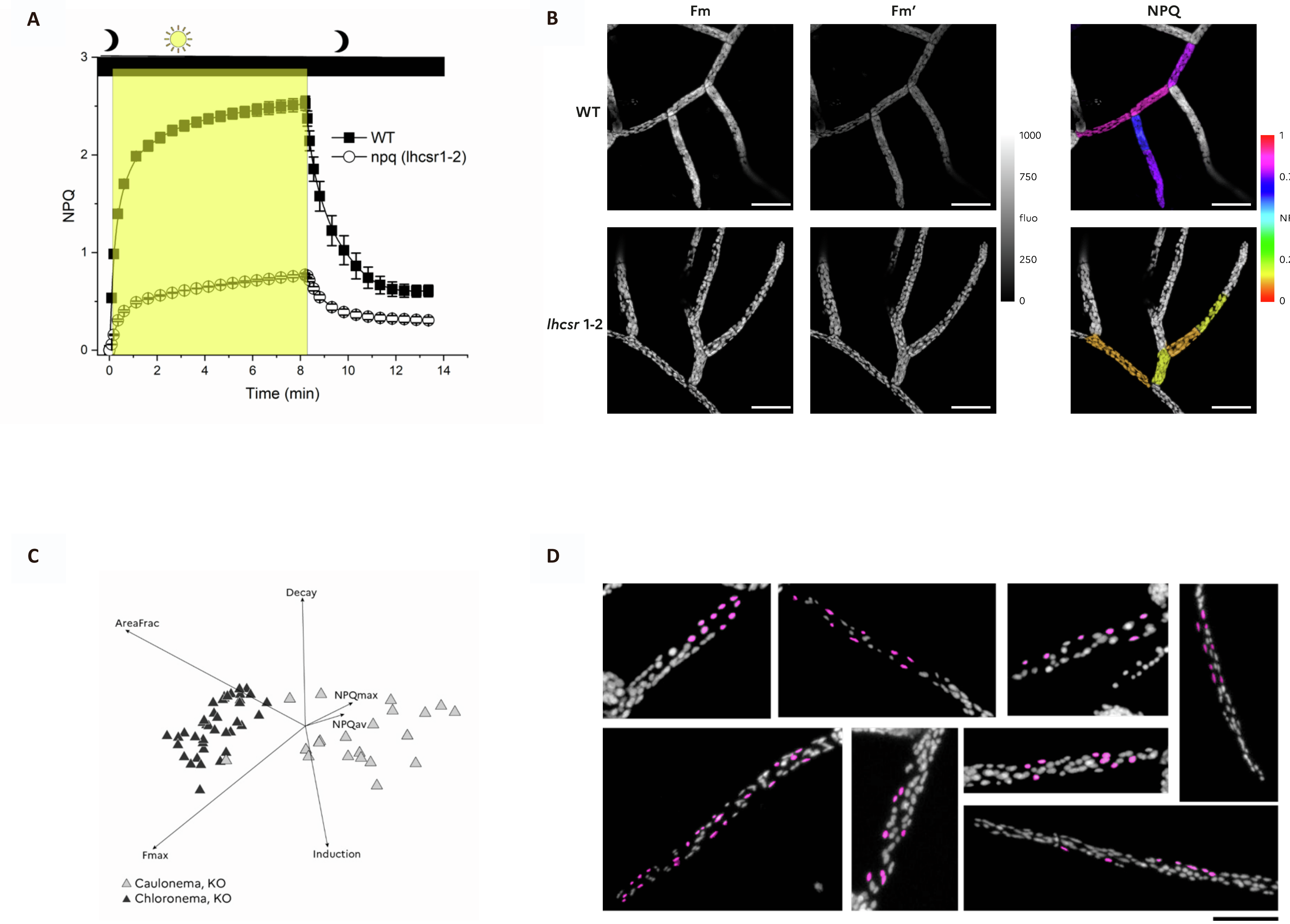

Figure S3 (related to figure 2-3): NPQ analysis of *P. patens* cells. (A), NPQ in *P. patens*, measured with a chlorophyll fluorescence imaging camera. WT (black squares) and *lhcsr1-2 (empty circles) P. patens* protonema were exposed to saturating light intensity (yellow overlay, 500 µmols photons m^{-2} s^{-1}) for 8 min before measuring photosynthetic parameters using a speedzen Chl fluorescence imaging setup (JBeambio, France).[47] Same symbols and box colours as in Fig 2D. **(B)**, Comparison between WT cells and mutant cells with reduced NPQ capacity (*lhcsr1-2* KO) Fm and Fm' represent the maximum fluorescence under dark condition and under actinic light respectively (grayscale). NPQ was calculated from Fm and Fm' and is visualised by artificial colour only for the analysed cells of the WT and mutant protonema. Scale bars 50µm. **(C)**, PCA analysis of NPQ features in the *P. patens lhcsr1-2* KO-mutant: second and third components**.** Second and third components of a PCA realised on 63 *P. patens lhcrs1/2* KO cells (solid symbols chloronema, open symbols: caulonema). The first two components represent roughly 88% of the variance and the first three components represents more than 94% of the variance (Supplementary Table 2). **(D)**, NPQ analysis of *P. Patens* single chloroplasts. Among the numerous plastids of *P. patens* protonema, some single chloroplasts (highlighted in pink) were selected because they never overlap with each other during the confocal microscope acquisition. Chloroplasts movements were tracked manually and fluorescence values were calculated in the dark and after 6 min of light exposure (500 µmol photons m^{-2} s^{-1}). Scale bar: 50 µm.

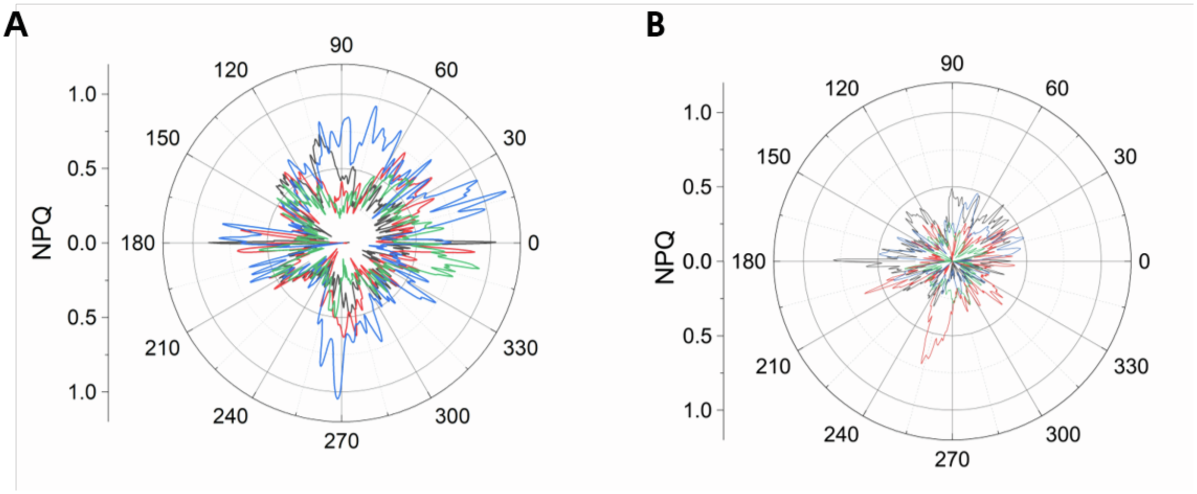

Figure S4 (related to Figure 4): subcellular analysis of NPQ in acantharians. Radar plot of the NPQ in large **(A)** and small **(B)** symbiotic microalgae. NPQ was calculated from images acquired in the dark and after exposure to actinic light (500 µmol photons m^{-2} s^{-1}) for 15 minutes. Different colours represent different phaeocystis cells.

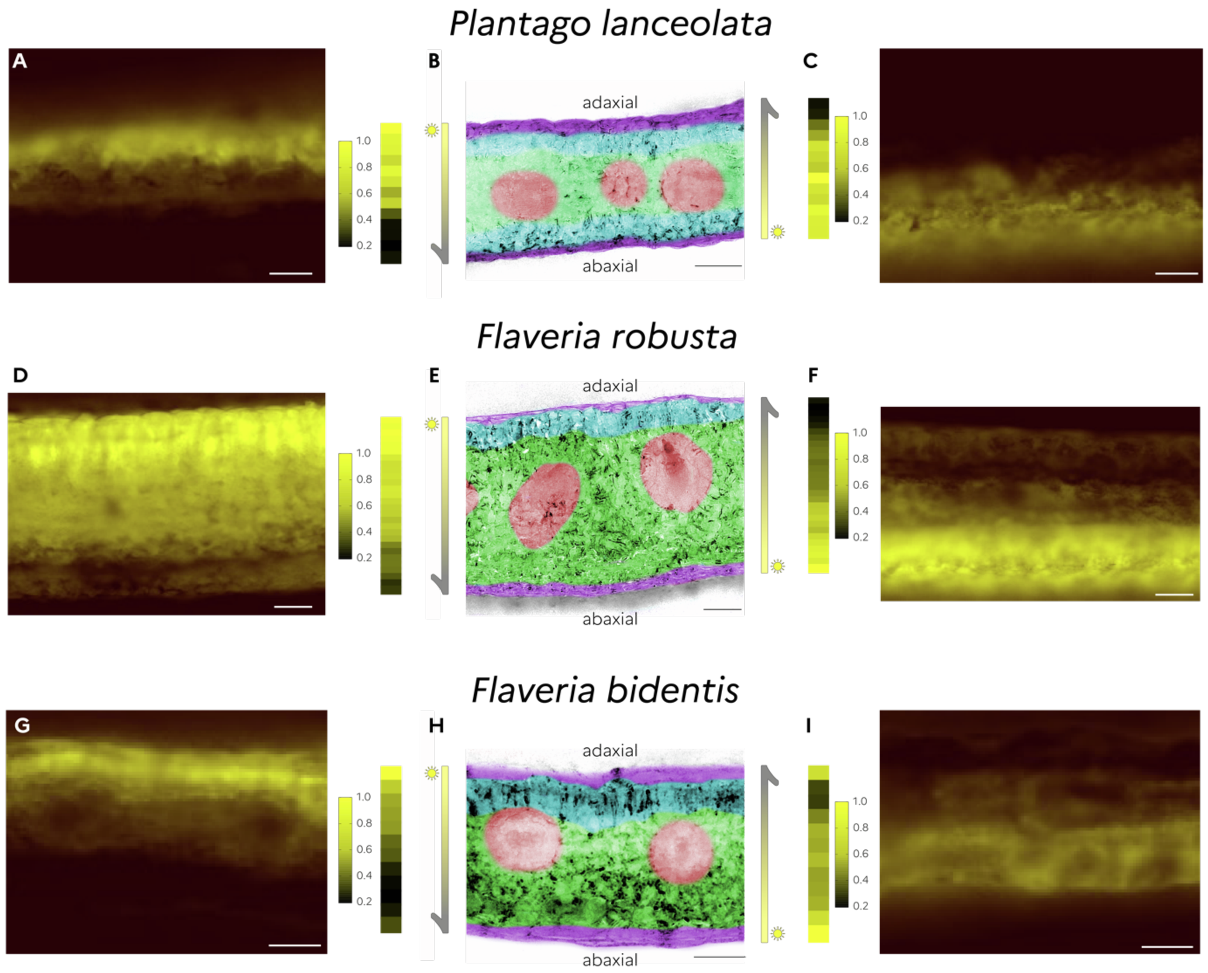

Figure S5 (related to Figure 5): Light gradients inside leaves. Light gradients are visualised by the difference in chlorophyll fluorescence (figured as yellow in artificial colour) intensity upon illumination in the adaxial to abaxial side (bottom to top –panels **A**, **D** and **G**) and abaxial to adaxial (bottom to top –panels **C**, **F** and **I**) for a representative cross section of leaves with different anatomies (panels **B**, **E** and **H** Scale bar : 100µm). The following colours were used to highlight the different leaf tissues: purple: epidermis; blue: palisade parenchyma; green: spongy parenchyma; red: vascular tissue

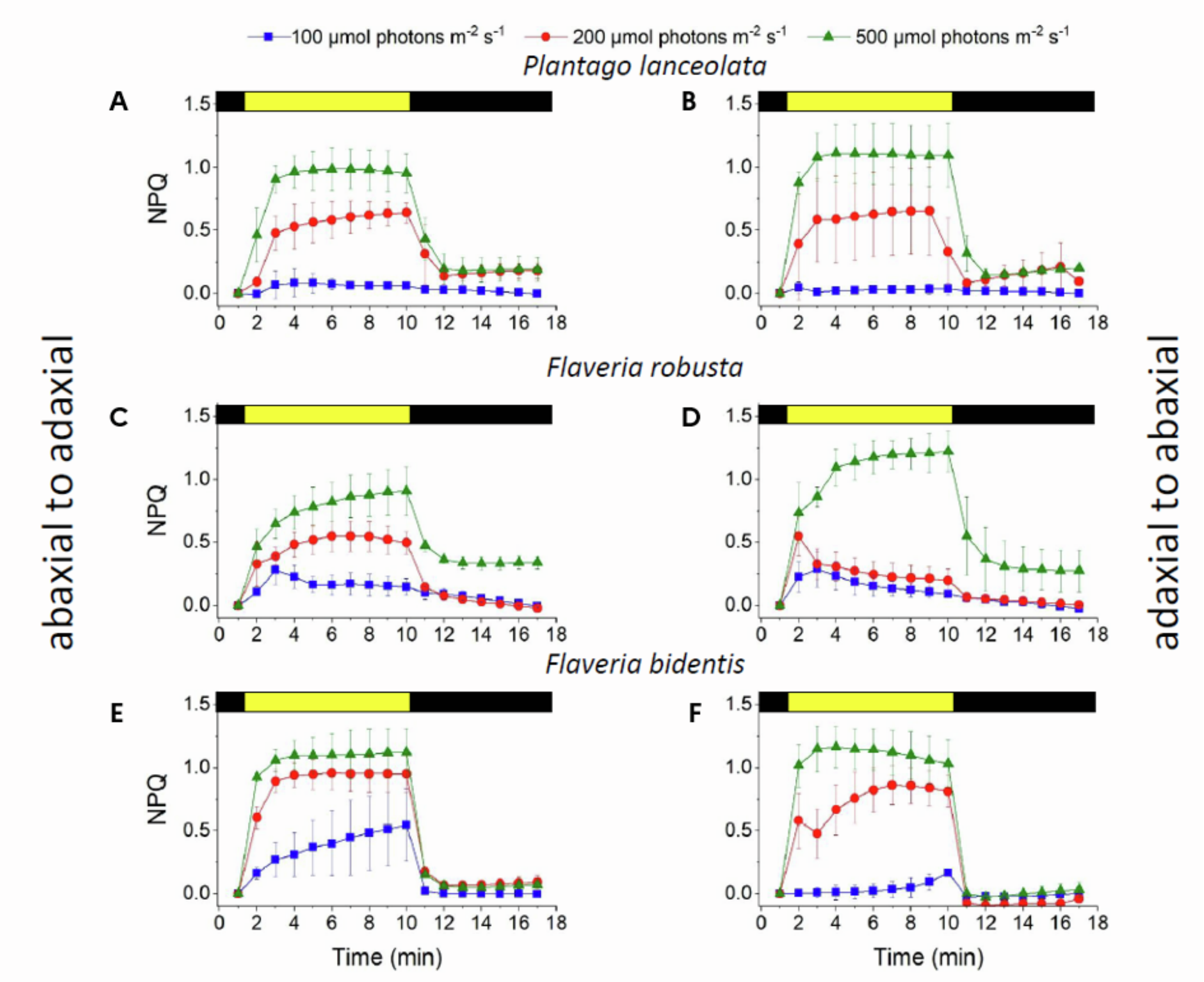

Figure S6 (related to Figure 5): NPQ features are modulated by leaves architectures. NPQ measured upon illumination in the adaxial to abaxial side (top to bottom –panels **A**, **C** and **E**) and abaxial to adaxial (bottom to top – panels **B**, **D** and **F**) for three leaves with different anatomies as presented in Fig. 4. Shown are means ± SDs (n=3)

	Comp. 1	Comp. 2	Comp. 3	Comp. 4	Comp. 5	Comp. 6
Standard deviation	1.9182243	1.2630922	0.60785746	0.45836413	0.35531842	0.138470303
Proportion of Variance	0.6132641	0.2659003	0.06158178	0.03501628	0.02104186	0.003195671
Cumulative Proportion	0.6132641	0.8791644	0.94074619	0.97576247	0.99680433	1.000000000

Supplementary Table 1 (related to Figure 3): Correlation table of the six observed variables

	NPQmax	NPQav	Decay	Induction	AreaFrac	Fmax
NPQmax	1.00000000	0.97681818	0.8537735	0.812678625	- 0.070494246	0.2514870
NPQav	0.97681818	1.00000000	0.8392191	0.837617127	- 0.020657062	0.2833078
Decay	0.85377353	0.83921910	1.0000000	0.701930935	0.195843670	0.3648589
Induction	0.81267862	0.83761713	0.7019309	1.000000000	0.009117372	0.3559048
AreaFrac	-0.07049425	-0.02065706	0.1958437	0.009117372	1.000000000	0.6719592
Fmax	0.25148703	0.28330778	0.3648589	0.355904772	0.671959243	1.0000000

Supplementary Table 2 (related to Figure 3): Principal Component Analysis results.

	1st Comp	2nd Comp	3rd Comp	4th Comp	5th Comp	6th Comp
NPQmax	24.4496826	3.7675641	0.9885236	3.6558842	14.9788645	52.15948102
NPQav	24.8978544	2.5525258	0.2549017	0.2827022	25.9588445	46.05317140
Decay	22.5153461	0.0140642	29.3140005	3.7408834	43.5351179	0.88058787
Induction	21.4494317	0.8431956	26.1007806	42.9901977	8.2970322	0.31936221
AreaFrac	0.6559251	53.9600310	16.4085692	21.8731751	6.5749696	0.52732993
Fmax	6.0317601	38.8626193	26.9332244	27.4571575	0.6551713	0.06006757

Supplementary Table 3 (related to Figure 3): Contribution table of the six observed variables to the construction of the six components. For example, the first component is constructed with a contribution of NPQmax with 24.5 %, NPQav with 24.9%, Decay with 22.5% and Induction with 21.5%
