# Supplementary material for: Tailoring confocal microscopy for real-time analysis of photosynthesis at single-cell resolution
Source: Cell Rep Methods. 2023 Aug 28;3(9):100568. doi: 10.1016/j.crmeth.2023.100568 (PMC10545909; doi:10.1016/j.crmeth.2023.100568)
Supplement: Document S2. Article plus supplemental information [file mmc5.pdf]

Cell Reports
Methods

Article

Tailoring confocal microscopy for real-time analysis of photosynthesis at single-cell resolution

Graphical abstract

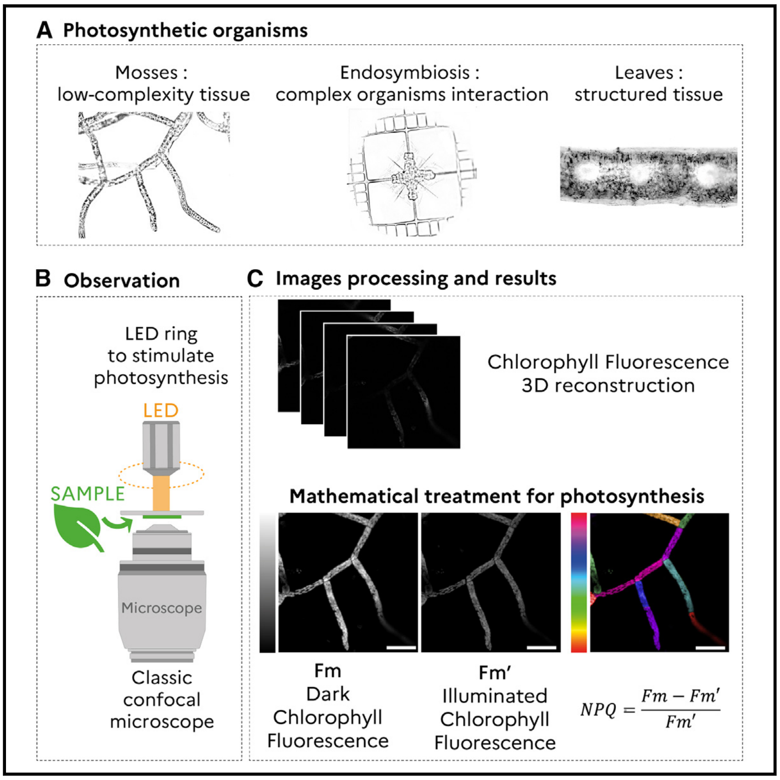

Highlights

- Real-time assessment of chlorophyll fluorescence at confocal microscopy resolution
- Study of photosynthesis at single-cell resolution within a living tissue/organism
- Relationship between photosynthetic features and cell type or morphology
- Mathematical modeling of photosynthesis in a cell volume 3D space

Authors

Mattia Storti, Haythem Hsine, Clarisse Uwizeye, ..., Gilles Curien, Giovanni Finazzi, Dimitri Tolleter

Correspondence

giovanni.finazzi@cea.fr (G.F.),
dtolleter@gmail.com (D.T.)

In brief

Storti et al. develop a method to study photosynthesis at single-cell resolution using confocal microscopy. They use this approach to examine photosynthesis specialization in different tissues and organisms and to understand the relationship between light penetration and photoprotection.

Storti et al., 2023, Cell Reports Methods 3, 100568
September 25, 2023 © 2023 The Authors.
https://doi.org/10.1016/j.crmeth.2023.100568

CellPress

Cell Reports Methods

CellPress
OPEN ACCESS

Article

Tailoring confocal microscopy for real-time analysis of photosynthesis at single-cell resolution

Mattia Storti,[1] Haythem Hsine,[1] Clarisse Uwizeye,[1] Olivier Bastien,[1] Daniel P. Yee,[1,2] Fabien Chevalier,[1] Johan Decelle,[1] Cécile Giustini,[1] Daniel Béal,[3] Gilles Curien,[1] Giovanni Finazzi,[1,*] and Dimitri Tolleter[1,4,*]
[1]Grenoble Alpes University, CNRS, CEA, INRAE, IRIG-LPCV, 38000 Grenoble, France
[2]Cell Biology and Biophysics Unit, European Molecular Biology Laboratory, 69117 Heidelberg, Germany
[3]JBeamBio, 17000 La Rochelle, France
[4]Lead contact
*Correspondence: giovanni.finazzi@cea.fr (G.F.), dtolleter@gmail.com (D.T.)
https://doi.org/10.1016/j.crmeth.2023.100568

MOTIVATION Visualizing photosynthetic responses in 3D is essential for understanding most acclimation processes, as light changes within photosynthetic tissues as it penetrates the absorbing/diffusing layers of the cells. To achieve this goal, we developed an imaging workflow merging confocal microscopy and saturating pulse chlorophyll fluorescence detection. This method applies to samples characterized by increasing complexity, and its simplicity will contribute to its widespread use in plant and microalgae photoacclimation studies.

SUMMARY

Photoautotrophs' environmental responses have been extensively studied at the organism and ecosystem level. However, less is known about their photosynthesis at the single-cell level. This information is needed to understand photosynthetic acclimation processes, as light changes as it penetrates cells, layers of cells, or organs. Furthermore, cells within the same tissue may behave differently, being at different developmental/physiological stages. Here, we describe an approach for single-cell and subcellular photophysiology based on the customization of confocal microscopy to assess chlorophyll fluorescence quenching by the saturation pulse method. We exploit this setup to (1) reassess the specialization of photosynthetic activities in developing tissues of non-vascular plants; (2) identify a specific subpopulation of phytoplankton cells in marine photosymbiosis, which consolidate energetic connections with their hosts; and (3) examine the link between light penetration and photoprotection responses inside the different tissues that constitute a plant leaf anatomy.

INTRODUCTION

Photosynthesis is a major bioenergetic process in the biosphere. It feeds most of the food chains on Earth and is responsible for substantial sequestration of CO_2 via the biological pump. The efficiency and regulation of this process is usually assessed *in vivo* by measuring chlorophyll (Chl) fluorescence,[1] i.e., the fraction of absorbed light that is re-emitted in the near-infrared region of the spectrum. Chl fluorescence can be analyzed at different scales: from the cellular level using microscopes to organs/organisms using infrared cameras or even at larger scales (ecosystems/planetary) using satellites.[2–4] Those approaches all suffer from a similar limitation, namely the detection of images in two dimensions only. However, significant changes are expected within the 3D volume of phototrophs, because the color and intensity of light vary according to its penetration into the absorbing/diffusing layers of photosynthetic cells.[5] So far, theoretical approaches have been used to extrapolate data obtained from a surface (leaf, canopy, or ocean) to a volume, by modeling light penetration.[6] In a few cases, the responses of a photosynthetic tissue (e.g., a leaf) to different colors of light (blue, green, and red) were compared to highlight the effect of different light penetrations on photosynthesis.[7–9] Both approaches have limitations as they do not rely on direct assessment of photosynthesis inside an intact photosynthetic tissue/organ. Moreover, monitoring fluorescence changes in two dimensions does not allow detecting fluorescence of a single plastid, because these organelles move inside cells.[10–12] To overcome this difficulty, we have explored the potential of a Chl fluorescence imaging approach that combines the spatial resolution of a confocal microscope with the reliability of the saturation pulse

This is an open access article under the CC BY license (http://creativecommons.org/licenses/by/4.0/).

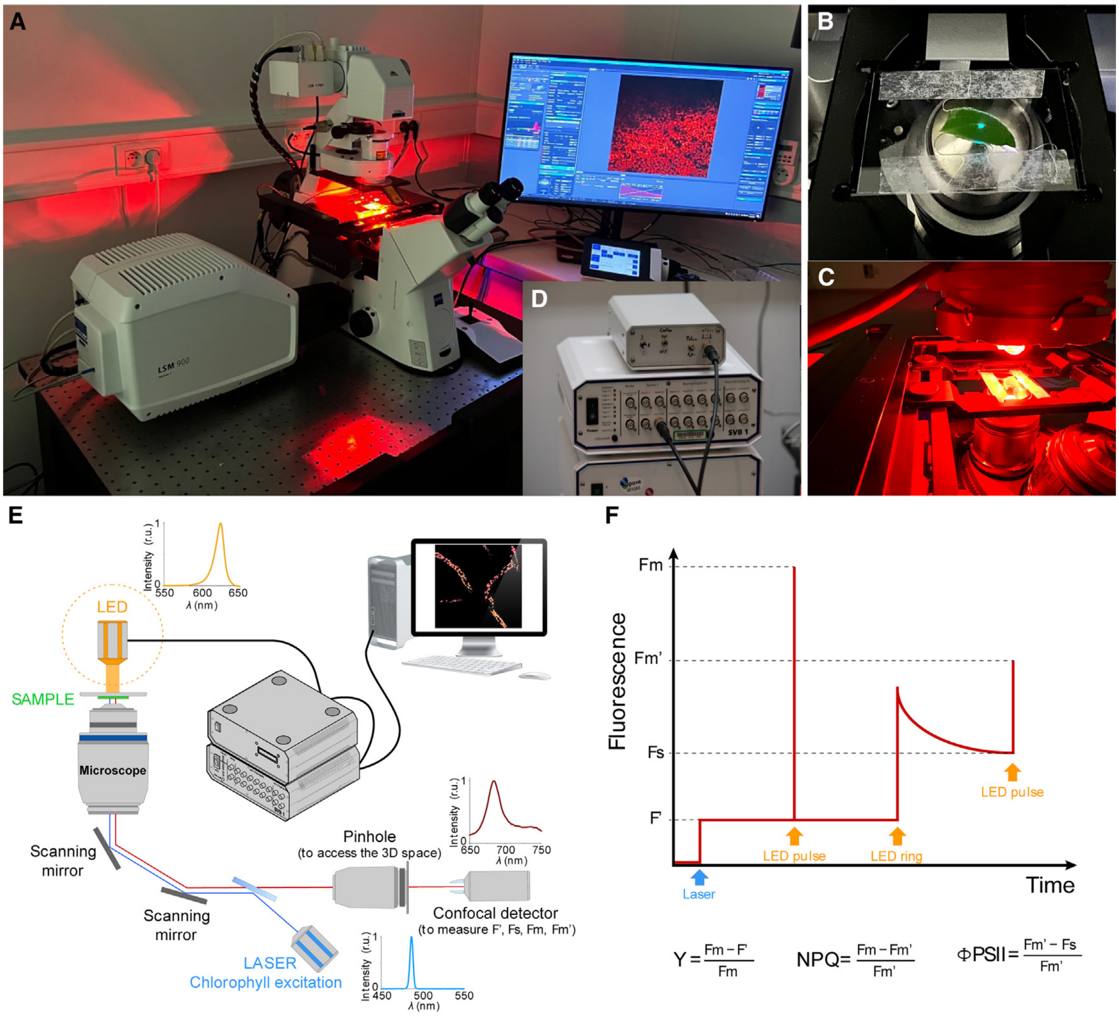

Figure 1. 3D-Pulse fluorimeter imaging setup

(A–D) Global view of the setup. (B) Close-up on the sample (here a piece of leaf) with the confocal laser on showing the small portion of the sample illuminated. (C). Close-up on the same sample with the actinic light on, showing an area of illumination larger than the sample. (D) Close-up of the control box and connections to the confocal system.

(E) Scheme of the customized confocal microscope includes an orange LED array controlled by the Zen software via the SVB1 module plus a homemade controller to deliver actinic light and saturating pulses.

(F) Switching on and off the light pulses via the "experimental design" routine (and manual switching on and off the actinic light) allows evaluating relevant photosynthetic parameters.

method.[13] The latter approach has been particularly successful in assessing relevant photosynthetic parameters (the quantum yield of photosystem [PS] II in the dark [Fv/Fm] and in the light [ΦPSII]) and the thermal dissipation of excess excitation energy (NPQ) to study CO_2 assimilation capacity,[14] plant acclimation to the environment, and stress responses.[15] We show that the 3D saturating pulse confocal setup provides unique physiological information concerning photoprotection in biological samples characterized by increasing complexity: (1) heterogeneous responses of single chloroplast/cell in mosses, which contain multicellular and partially differentiated tissues, (2) complex relationships in photosymbiosis between eukaryotic host cells and symbiotic microalgae at different developmental stages, (3) the link between leaf architecture and photoprotection in vascular plants.

RESULTS

Combining a saturating pulse method with a confocal microscope

To develop a 3D saturating pulse confocal (hereafter 3D-Pulse fluorimeter, Figure 1A), we equipped a Zeiss LSM900 inverted confocal microscope (for alternative systems, see Table 1) with an additional red light LED source (λ = 630 nm, full width-half maximum 18 nm) placed in front of the microscope objective (Figures 1B–1E). The LEDs deliver, on the entire sample (Figure 1C), short and intense pulses (2,000 μmol photons m^{-2} s^{-1}) to saturate PSII and thus achieve maximum Chl fluorescence emission, Fm.[14,16] The LEDs also provide continuous actinic light of adjustable intensity (actinic light), to achieve

Table 1. Equipment needed to adapt our method on different confocal systems

Brand (by alphabetic order)	Confocal system	Electronic interface	Software module
Leica	STELLARIS series	trigger box 158004760 (ref. Leica)	LAS X live data mode 158203201 (ref. Leica)
Nikon	A1, AX series	Ti2 controller (Digital I/O)	NIS-Elements advanced interpreter (MQS42510)
Olympus	FLUOVIEW series	FV30-analog	Fluoview 3000
Zeiss*	LSM series*	SVB1 module*	“experimental design” routine of the ZEN software (v.3.0)*

Asterisks (*) indicate the systems used in this study.

steady-state (Fs) fluorescence (Figure 1F). Both the pulses and the continuous light delivered by the LED array are operated by a home-built control box (Figures 1D and S1). The blue laser (λ = 488 nm) of the confocal is used as the “measuring light” in the saturation pulse method[13] to image Chl fluorescence (Figure 1E), leading to a parameter hereafter called F' (Figure 1F). This parameter is close to the F0 parameter used in the saturating pulse method.[13] Both the blue laser, the acquisition (on a predefined z stack) and the LED control box are controlled by the confocal software Zen (version 3.0), through the “experiment designer” routine. We used the Fiji software[17] to analyze the datasets and treated the images as follows: 3D time series acquisitions (xyzt) were converted to 2D images (xyt) in a way that preserves original fluorescence values (“sum slices” function). The latter can be calculated for every time point measuring the “mean gray value” of regions of interest (ROIs) (chloroplasts, cells, and tissues) and subtracting the background fluorescence (i.e., the signal measured in an empty ROI located near the measurement region). Indeed, background fluorescence is also affected by external light source (Figure S2). When needed, image segmentation was done with the 3D Slicer software[18] to generate 3D models that gave information about the plastids volume. We calculated photosynthetic parameters from fluorescence values with the Origin software (Microcal).

We first validated the 3D-Pulse fluorimeter on a photosynthetic organism having a relatively simple structure, the juvenile gametophyte (protonema) of the moss *Physcomitrium patens*. We noticed that the maximum photosynthetic capacity, here indicated by the PSII-related parameter Y = (Fm − F′)/Fm, decreased when the laser intensity increased. The Y decrease reflects the actinic effect of blue laser itself, which increases the F′ parameter. However, the lack of fluorescence quenching during exposure to multiple (four) laser pulses before the saturating pulse (Figure S2B) demonstrates that each measurement does not influence the subsequent one. At 0.3% laser intensity (i.e., 220 μJ cm^{-2}, Figures 2A and 2B), the F′, obtained in the presence of the blue laser illumination alone, reached the same level as Fm, which is the fluorescence intensity obtained by concomitant illumination with the blue laser and the saturating red pulse. This finding suggests that the blue laser alone, which is localized over a very small area, saturates photosynthesis (inducing Fm) even at relatively low intensities. We exploited this possibility to measure Fm and Fm’ without the saturating pulse protocol, and therefore to calculate NPQ, which is readily estimated from these two parameters (Figure 1F). On the other hand, the relative PSII yield can be evaluated using a sub-saturating laser intensities (Figure 2B).

The 3D-Pulse fluorimeter was fast enough to measure the kinetics of NPQ onset (when the actinic light was switched on) and its relaxation in the dark (Figure 2C; Video S1). We could also detect changes in NPQ amplitude as a function of actinic light intensity, as well as a transient NPQ during exposure of the dark-adapted protonema filaments to low light intensity (Figure 2C, dash and dotted line). This transient NPQ reflects the link between activation of CO_2 assimilation and photoprotective responses: at the beginning of illumination, when CO_2 assimilation is largely inactive, part of the absorbed light is dissipated. Conversely, most of the absorbed photons are drained to CO_2 assimilation in steady state (when the Calvin-Benson-Bassham cycle is fully active) and thus NPQ disappears.[19] Finally, we could easily differentiate NPQ features of a WT and a mutant strain with reduced NPQ capacity (due to knocking out of the NPQ effector proteins LHCSR1 and LHCSR2) (Figures 2D, S3A, and S3B; Video S2).[20] We noticed that NPQ was lower in the 3D-Pulse fluorimeter than in a conventional 2D imaging fluorimeter equipped with similar light sources and intensities (Figure S3A). While this difference could be caused by the lower amount of actinic light delivered inside the tissue (where the 3D-Pulse fluorimeter measures), the kinetic features that we observed (Figures 2C and 2D) are consistent with the occurrence of a genuine quenching process (NPQ)[21] in all the investigated samples.

Photoprotective responses in non-vascular plants

We explored the possibilities offered by the 3D-Pulse fluorimeter to study cellular and subcellular NPQ responses in the two cell types that constitute the protonema of *P. patens*: the caulonema and the chloronema. The former has longitudinally elongated cells involved in propagation and nutrient acquisition, the latter has chloroplast-rich cells, usually considered as the photosynthetic part of the moss protonema (Figure 3A).[22] As plastids move inside the cell,[10,23] they tend to leave the field of observation in a conventional microscope during the relatively long time required for NPQ development (Video S3). Instead, we could follow plastid responses within the entire volume of caulonema and chloronema cells with the 3D-Pulse setup (Video S1), visualize Chl fluorescence (Figure 3B), cell fraction occupancy (Figure 3C), and quantify NPQ capacity (Figure 3D) of single cells and plastids. A principal-component analysis of 175 mutant and WT cells allowed to distinguish four classes (Figure 3E; Tables S1–S3): the WT (circles) and the NPQ mutant *lhcsr1-2* (triangles) were separated based on their NPQ capacity, while the two cell types (black, chloronema; gray, caulonema) could be differentiated because of their plastid cell density. A more refined analysis (WT, Figure 3F, see also

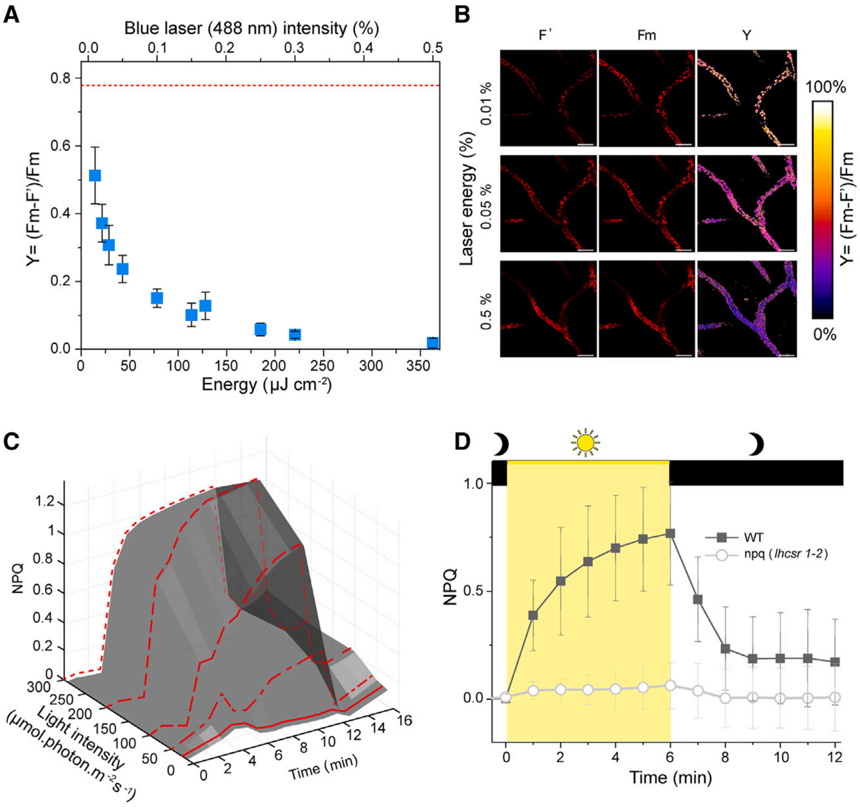

Figure 2. Validation of the 3D-Pulse fluorimeter

(A) The apparent quantum yield of photosystem II (Fm-F′)/Fm is lower than values measured with a standard Chl fluorescence imaging camera (red dashed line), and decreases as a function of the energy of the confocal laser power (blue squares, n = 7 cells average ± SD).

(B) Representative Chl fluorescence images (red) used to calculate Y (artificial color). Scale bar, 50 µm.

(C) NPQ changes as a function of the light intensity (25, 50, 100, 200, and 500 µmol photons m^{-2} s^{-1}). Representative traces of an experiment repeated 5 times with similar results.

(D) NPQ features in a WT (solid symbols, average of 108 cells ± SD) and mutant (open symbols, average of 63 cells ± SD) with downregulated NPQ capacity. Dark box, actinic light off; yellow box, actinic light on (500 µmol photons m^{-2} s^{-1}).

Figure S3C for the *lhcsr1-2* mutant) revealed subtle heterogeneity in NPQ responses in both caulonema and chloronema, which we could interpret based on 3D imaging. We identified heterogeneous NPQ responses at the cell level (Figure 3G), which account for most of the above-mentioned heterogeneity. Conversely, single plastids (Figure S3D) within a given cell behave homogeneously (Figure 3H): cells with high photoprotective responses contain plastids with high NPQ capacity, while cells with low photoprotection have plastids with low NPQ. Such variability in NPQ responses at the cell level probably reflects the different physiological state of cells that continuously regenerate during protonema development. While it is relatively easy to distinguish caulonema from chloronema in the complex matrix of the protonema, it is difficult to attribute more specific cellular characteristics (such as their age), which certainly have an impact on photosynthetic behavior.

Probing heterogeneous photosynthetic activity inside a complex, photosymbiotic organism

We further investigated the ability of the 3D-Pulse fluorimeter to link NPQ responses to different physiological states focusing on photosymbiosis, a common lifestyle in oceanic plankton between symbiotic microalgae and unicellular eukaryotic hosts.[24–28] Inside hosts (acantharians), symbiotic microalgae (the haptophyte *Phaeocystis cordata*, Figure 4A) undergo progressive morphological and metabolic changes (i.e., multiplications of plastids). Therefore, a single host cell contains a mix of newly engulfed/small symbionts with two plastids, and of larger (presumably older) ones, with up to 60 plastids as revealed by focused ion beam scanning electron microscopy imaging.[29,30] Using confocal microscopy, we confirmed the algal heterogeneity in terms of cell volume occupied by plastids (Figure 4B, histogram).[30] Moreover, we were also able to extract quantitative photosynthetic characteristics of individual microalgae inside the host upon segmentation and 3D reconstruction of their Chl fluorescence emission (Figure 4B).

Relating plastid volume heterogeneity to single-cell NPQ responses revealed the existence of two symbiont populations: small algae (open symbols) exhibited lower NPQ (Figure 4C) and photosynthetic activity (assessed by the ΦPSII parameter, Figure 4D) than free-living cells in culture measured with the same 3D-Pulse fluorimeter (Figures 4C and 4D, blue bars). Conversely, larger symbionts (solid symbols) had a different trend: their NPQ was always lower, while ΦPSII was higher than in free-living cells. We interpret the lower NPQ but higher ΦPSII of larger symbionts as a signature of enhanced photosynthetic performance upon transformation of the alga inside the host, as reported previously.[29] The physiological activity of the small symbiont population has not been reported so far in photosymbiosis, likely because it represents a relatively small fraction of the symbiotic cells, difficult to observe with conventional Chl fluorescence imaging setups. Their photosynthetic features (concomitant decrease of photosynthesis and NPQ) are reminiscent of the ones observed in the diatom *Phaodactylum tricornutum*[31] and the green alga *Chlamydomonas reinhardtii*[32] upon mineral nutrient (Fe) limitation. We propose that this particular population comprises microalgae in the process of adapting to the trophic environment provided by the host (see discussion).

We investigated their NPQ features at the subcellular level. However, it was difficult to track single *Phaeocystis* plastids separately because these organelles are around 10 times smaller than the ones found, e.g., in plants and *P. patens*.[33,34] Moreover, they are

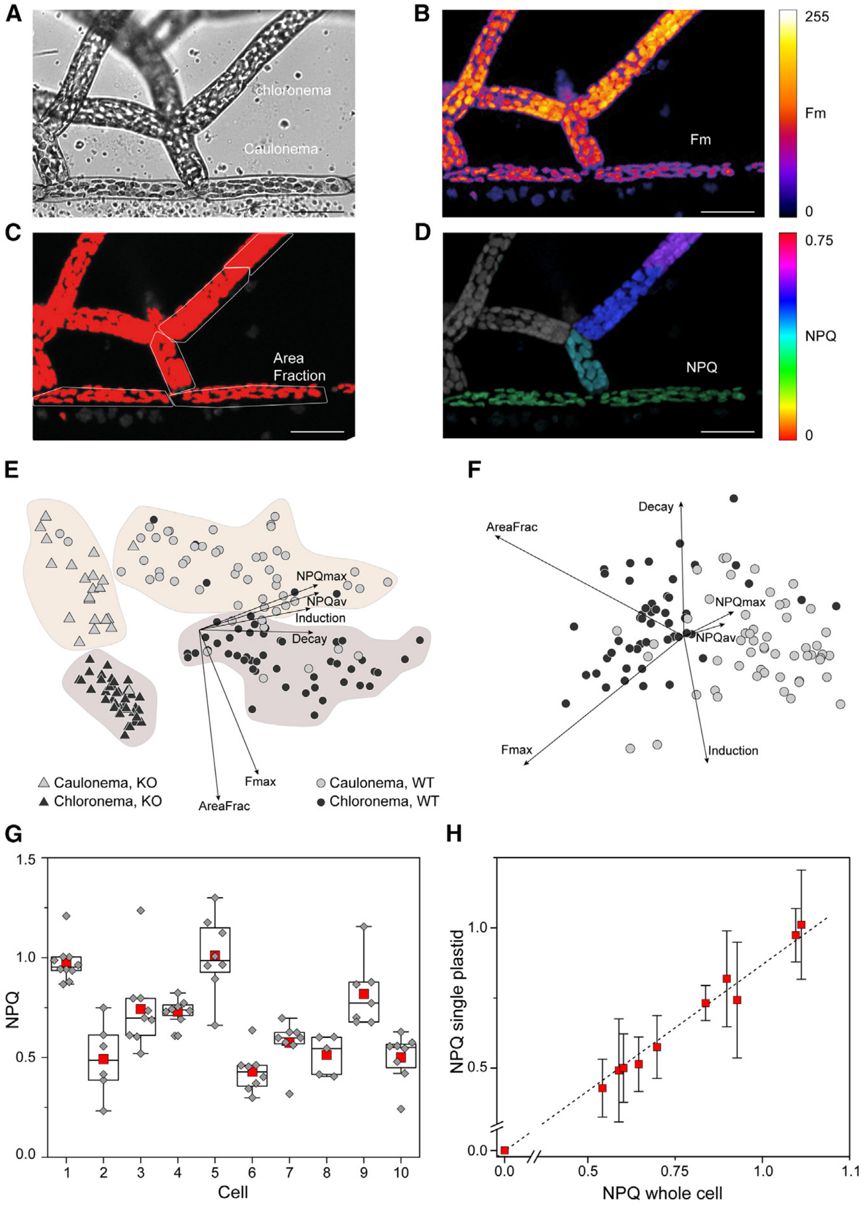

Figure 3. NPQ features in growing tissues of the moss *Physcomitrium patens*

(A) Bright-field image.

(B) Artificial color image of chlorophyll fluorescence.

(C) Fraction of the cells occupied by plastids (red).

(D) Artificial color image of NPQ in caulonema and chloronema cells of *P. patens* protonema. Scale bars, 50 μm.

(E and F) Principal-component analysis of 175 cells (black circles, chloronema WT; black triangles, chloronema *lhcsr1-2* KO; gray circles, caulonema WT; gray triangles, caulonema *lhcsr1-2* KO). (E) First and second components and (F) second and third components for WT cells. The first two components represent roughly 88% of the variance, while the first three components represent more than 94% of the variance (Table S1).

(G) NPQ is heterogeneous in *P. patens* cells. Red squares, mean of NPQ values of all plastids inside the same cell (gray diamonds; boxes, P25 and P75; whiskers, outliers; black line, median.

(H) Plastid vs. cell NPQ relationship reveals that plastids (average of 5–10 ± SD) behave homogeneously inside a given cell.

very densely packed[30] and thus cannot be easily separated based on confocal images. Thus, we developed an alternative data analysis approach: we first selected a 2D slice from the plastid 3D volume. Chl fluorescence values were calculated along the radius of a circular surface, approximating cell area. This was done for acquisitions of dark-adapted samples and for cells exposed to 10 min of illumination to evaluate the Fm, Fm', and NPQ ((Fm – Fm')/Fm') parameter. We repeated this calculation on different radii (separated by 5°) to infer homogeneous/heterogeneous NPQ responses inside the cells. Thanks to this approach, we could highlight heterogeneous NPQ values in both large (Figure 4E) and small (Figure 4F) symbionts (see also Figure S4), only using a single NPQ measurement. This result suggests that plastids of photosymbiotic cells develop NPQ responses in a rather heterogeneous manner when compared, e.g., to mosses.

Vascular plant NPQ is regulated by light channeling throughout anatomically diverse leaf architectures

Finally, we exploited the 3D-Pulse fluorimeter to investigate fluorescence responses in highly complex photosynthetic architectures: vascular plant leaves. These highly efficient machineries are composed of millions of cells, receiving variable light intensity depending on their position inside the organ. Cells on the surface receive more photons than cells inside the leaf, resulting in a light gradient.[35] The light gradient in turn leads to a different extent of saturation of photosynthesis, which we inferred via the amount of light in excess dissipated via NPQ. Thanks to high resolution of the 3D-Pulse fluorimeter, and the penetration of the blue laser, we could measure NPQ in mesophyll cells around 80 μm under the epidermis (vertical bars with blue arrows in Figure 5) of a leaf exposed to the red actinic light on the opposite side of the detection. In a monocotyledon (*Plantago lanceolata*), we recorded similar NPQ responses on the adaxial and abaxial sides (Figures 5A and 5C) consistent with the observation of a symmetrical leaf morphology (Figure 5B), and thus of similar light gradients in both directions (Figure S5).

Conversely, we observed heterogeneous NPQ responses in dicotyledons (*Flaveria robusta* and *Flaveria bidentis*), which harbor an asymmetric mesophyll organization (Figures 5E and 5H): on the adaxial face, the palisade parenchyma (blue) under the epidermis (purple), is made up of elongated photosynthetic cells arranged perpendicular to the leaf surface. On the opposite

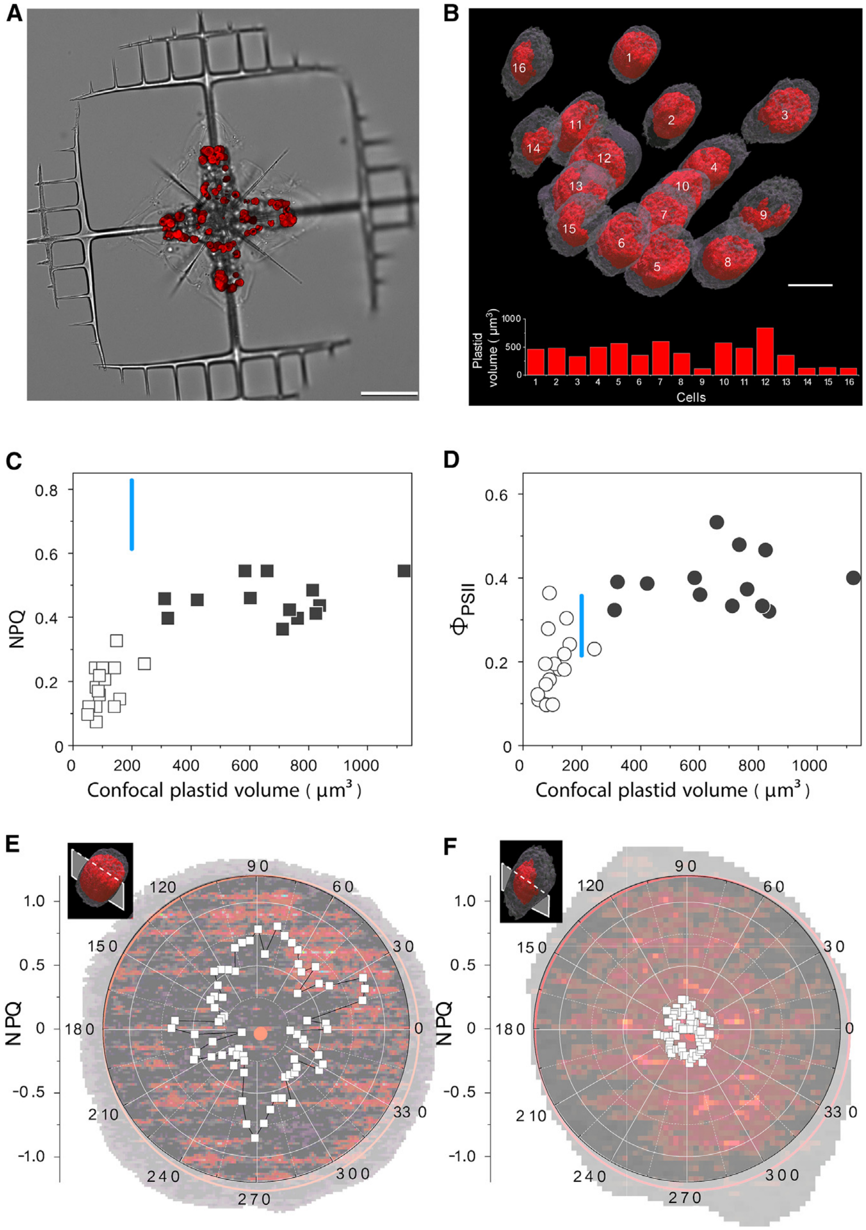

Figure 4. Photosynthetic features in a planktonic photosymbiosis

(A) Bright-field (gray) and Chl fluorescence (red) images overlaid from a host acantharian cell harboring symbiotic microalgae (the haptophyte *Phaeocystis*). Scale bar, 50 µm.

(B) 3D reconstruction of plastid chlorophyll fluorescence inside 16 microalgae (top) reveals large differences in their plastid volumes (bottom). Scale bar, 10 µm.

(C and D) Single-cell analysis reveals the existence of two symbiont populations having different photoprotective responses (NPQ) and photosynthesis (ΦPSII); in white the small symbiotic cells, in black the large symbiotic cells. Blue bars, NPQ and ΦPSII in free living *P. cordata* cells (n = 15 ± SD).

(E and F) Radar plot of the NPQ (white squares) in large (E) and small (F) symbiotic microalgae. NPQ was calculated from images acquired in the dark and after exposure to actinic light (500 µmol photons m^{-2} s^{-1}) for 15 min. Representative traces of an experiment performed on eight cells (E) and five cells (F), respectively (Figure S4).

abaxial side, this layer is replaced by the lacunar parenchyma (green), which occupies a large part of the leaf surrounding the vascular tissues (red) and consists of more irregular cells and large intercellular spaces to promote gas circulation and storage.

In both Flaveria species, NPQ at non-saturating light intensities was higher when actinic light was provided from the adaxial side to abaxial side (top to bottom, Figures 5D and 5G) compared with actinic light provided from the abaxial to the adaxial side (bottom to top, Figures 5F and 5I). By combining our 3D-Pulse fluorimeter derived findings with a more "classic" approach (measuring light penetration gradients inside a leaf,[6] Figure S5) we interpreted these data as follows: thanks to channeling through the parallel cell layers of the palisade mesophylls, light better penetrates the leaf in the adaxial to abaxial direction (Figure S5). Therefore, it saturates photosynthesis at a lower photon density and induces high photoprotective responses (NPQ) (Figure S6). Conversely, illumination in the abaxial to adaxial direction appears to favor light scattering from randomly oriented spongy cells. This phenomenon decreases photon penetration, lowers excess light, and therefore NPQ. Consistent with this conclusion, we observed that the dissymmetry of the NPQ was exacerbated in *F. bidentis*, where the steepness of the light gradient is higher due to a reduced thickness of the leaf (Figure S5). In agreement with this notion, differences in NPQ were erased when the light was increased to 500 µmol photons m^{-2} s^{-1}, an intensity that should over-saturate photosynthesis regardless of the direction of illumination. Hence, our method allowed characterizing *in situ*, without the use of models or assumptions, the effect of differential light penetration on photosynthesis.

DISCUSSION

In this work, we show that imaging Chl a fluorescence with a 3D saturating pulse confocal setup is well suited to image photosynthetic responses in three dimensions. Previous attempts to use a confocal microscope to measure photosynthesis[36,37] suffer from the limitations of using the confocal laser as the "measuring light," the "actinic light," and the "saturating pulse" of the saturating pulse approach. This choice implies that two different lights cannot be provided at the same time (unlike in the saturating pulse approach), thus hampering the accuracy in the

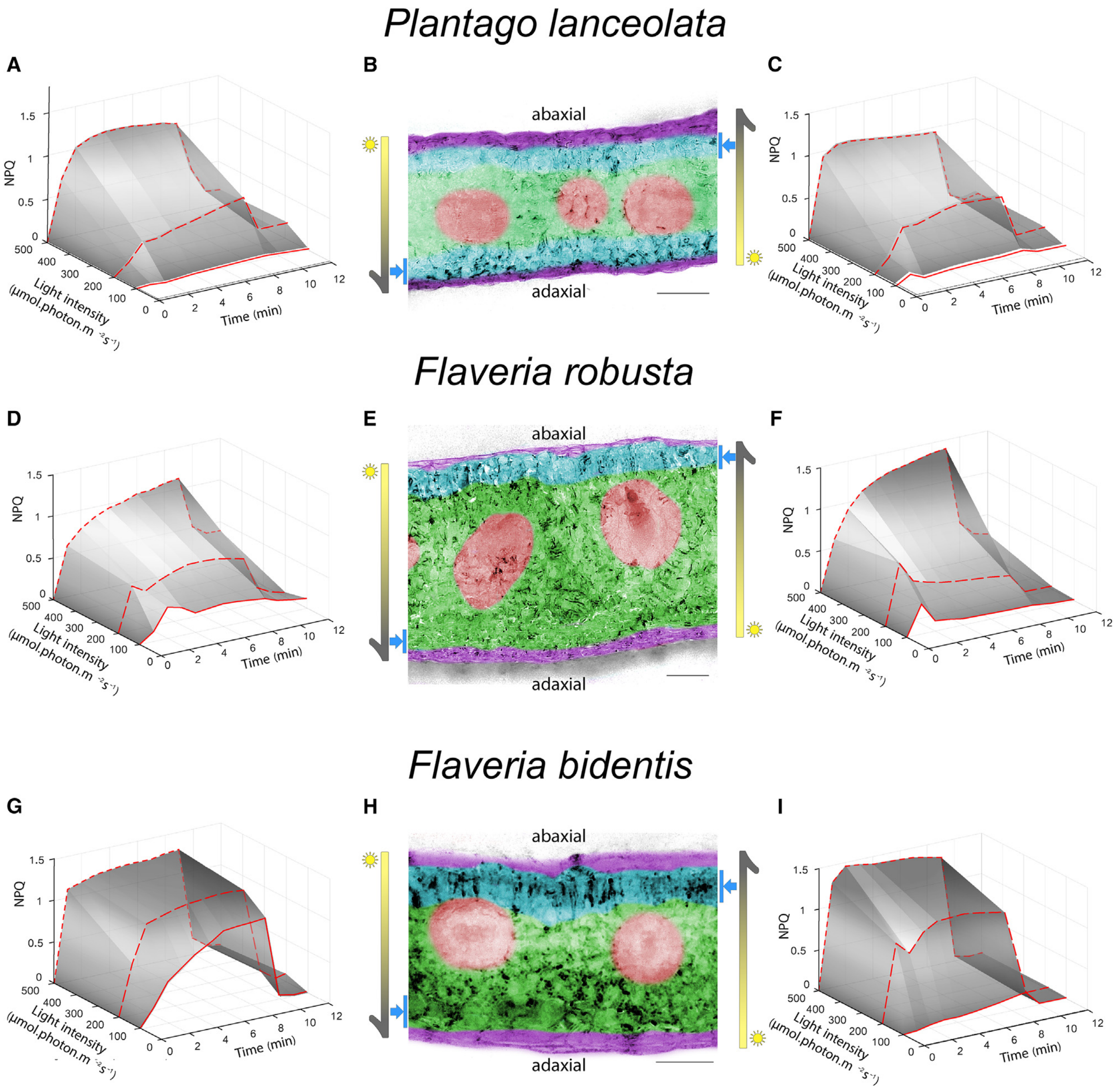

Figure 5. NPQ features are modulated by leaves' architectures

(A–I) NPQ measured upon illumination in the adaxial to abaxial side (top to bottom, A, D, and G) and abaxial to adaxial (bottom to top, C, F, and I) for three leaves with different anatomies. Representative picture of an experiment repeated 3–7 times with similar results. (B, E, and H) Artificial colors representation of the different leaf tissues: purple, epidermis; blue, palisadic parenchyma; green, spongy parenchyma; red, vascular tissue. Vertical bars with blue arrows represent the region imaged in NPQ experiments. Scale bars, 100 μm (gray).

determination of the photosynthetic parameters and the number of possible applications (e.g., discriminating the stomata from epidermis in plant leaves, assessing single plastid fluorescence transients).[36,37] Conversely, our setup combines the sensitivity and flexibility of the saturation pulse method with the 3D spatial resolution of confocal microscopy to investigate photosynthesis *in vivo* at the organ, cell, and subcellular levels. Previous work concluded that chloronema cells fix carbon within the protonema, while caulonema cells are mostly involved in nutrient propagation and acquisition.[38–40] Our analyses indicate that the photosynthetic differences between these two cell types cannot be attributed to the intrinsic properties of PSII since photosynthetic parameters derived from PSII analyses (NPQmax, NPQav, decay, and induction parameters) are similar. Instead, we confirm that the net amount of the photosynthetic machinery as shown by the cell fraction occupied by plastids (Figure 3F,

AreaFrac parameter) and the total Chl fluorescence emission (Figure 3F, Fmax parameter) is different for the two tissues. These differences, together with previously reported metabolic and transcriptomic ones in these two types of cells,[39,40] likely explain the difference between caulonema and chloronema in the global process of carbon fixation.

In acantharians, we identified a particular algal subpopulation characterized by very low photosynthetic performance and photoprotection capacity. In general, these two parameters show complementary responses: low photosynthesis results in high dissipation of excess light in the form of NPQ, whereas high photosynthesis leads to low dissipation of excess light. The latter behavior is indeed observed in the large cells (Figures 4C and 4D), which probably represent algae that have established fully metabolic connections with the host.[30] Conversely, the population with low photosynthetic performance is made up of small cells, probably still adapting to the host trophic environment.[30] Indeed, a concomitant decrease in photosynthesis and photoprotection responses (NPQ) has only been reported in microalgae exposed to mineral nutrient (Fe) limitation.[31,32] It is tempting to draw a parallel between this population, which likely represents a transient stage in the establishment of photosymbiosis, and the hypothetical early stage of endosymbiosis.[41–43]

Our analysis of leaf photosynthesis supports previous findings that light is differentially channeled through different leaf tissues.[6] However, we go one step further, showing that moderate light intensity, which is on average received by most leaves in a tree due to mutual shading, can saturate photosynthesis over the entire leaf section in asymmetric leaves (Figures 5E and 5F). This is, however, only true when photons are captured on the adaxial side, where the palisade parenchyma channels them toward the opposite side of the leaf (Figures 5D and 5G), the spongy parenchyma. Overall, these results confirm the notion that leaf photosynthesis is largely governed by its anatomical features[44] and further extends it to the cellular level.

Limitations of the study

Our 3D imaging approach has clear advantages over conventional PAM fluorometers and single-cell pulse-probe microscopes, since it allows (1) disentangling heterogeneous cellular responses within a tissue (Figure 3), (2) tracking photosynthetic changes during cell development (Figure 4), (3) assessing the relationship between leaf anatomy (asymmetric or symmetric) and photoprotection (Figure 5). Its 3D resolution allows following the movements of plastids during prolonged exposure to light (Videos S1 and S2) to monitor their physiological responses.

However, there is a main difficulty associated with this approach. The choice of the laser light intensity is essential to ensure correct measurements of the photosynthetic parameters. In our case, the maximum photosynthetic capacity (the "Y" parameter in Figure 1F) decreases as a function of laser intensity (Figure 2A), as PSII becomes inactive (light saturated). The PSII is completely saturated (i.e., the Y parameter goes to 0) at 0.3% of the maximum power, indicating that the laser is too intense. In this case, this difficulty could be alleviated by placing neutral filters between the laser and the sample to decrease the laser power. Other organisms (or setups) may have different responses to light, so an experiment similar to that shown in Figure 2A is recommended before undertaking NPQ measurements.

STAR★METHODS

Detailed methods are provided in the online version of this paper and include the following:

- KEY RESOURCES TABLE
- RESOURCE AVAILABILITY
  - Lead contact
  - Materials availability
  - Data and code availability
- EXPERIMENTAL MODEL AND SUBJECT DETAILS
  - Photosynthetic models used in this study
- METHOD DETAILS
  - Sample preparation
  - Confocal microscope setup
- QUANTIFICATION AND STATISTICAL ANALYSIS
  - PSII yield and NPQ calculation
  - 3D reconstruction and fluorescence integration
  - Principal components analysis (PCA)

SUPPLEMENTAL INFORMATION

Supplemental information can be found online at https://doi.org/10.1016/j.crmeth.2023.100568.

ACKNOWLEDGMENTS

The authors thank Prof. Alessandro Alboresi (University of Padova) for providing *P. patens* samples, Prof. Benjamin Stich (University of Düsseldorf) for providing seeds of Flaverias, Dr. Charlotte Lekieffre (CEA Grenoble) for technical help with acantharians, and the institutes that supported the collection of samples: EMBRC-France and the Laboratoire d'Océanographie de Villefranche-sur-Mer. We thank also Dr. Benjamin Bailleul, Dr. Bernard Genty, and Prof. David Macherel for their critical reading.

This project received funding from the European Research Council: ERC Chloro-mito (grant no. 833184) to F.C., J.D. M.S., and G.F. and the European Union H2020 Project BIOTEC-02-2019 GAIN4CROPS (grant no 862087) to G.C., D.T., and G.F. Research was also supported by a Défi X-Life grant from CNRS to G.F. The ANR-11-BTBR-0008 Océanomics, ANR-15-IDEX-02 GlycoAlps and "Origin Of Life" Cross Disciplinary Projects of the Univ. Grenoble Alpes, and ANR-17-EURE-0003 to O.B. J.D. and F.C. were supported by CNRS and ATIP-Avenir program. D.P.Y. was supported by the IDEX project of University of Grenoble alpes (International Strategic Partnerships) and EMBL. Funding by the LabEx GRAL (ANR-10-LABX-49-01), financed within the University Grenoble Alpes graduate school (Ecoles Universitaires de Recherche) CBH-EUR-GS (ANR-17-EURE-0003), is also acknowledged.

AUTHOR CONTRIBUTIONS

Conceptualization, G.F. and D.T.; methodology, M.S., D.B., D.T., and G.F.; image analysis, M.S., C.U., and H.H.; data analysis, M.S. and O.B.; investigation, M.S., H.H., G.F., and D.T.; resources, D.P.Y., F.C., C.G., and D.T.; writing – original draft, M.S., D.T., and G.F.; writing – review & editing, M.S., G.C., G.F., and D.T.; funding acquisition, G.F.; supervision, G.C., J.D., G.F., and D.T.

DECLARATION OF INTERESTS

The authors declare no competing interests.

CellPress
OPEN ACCESS

INCLUSION AND DIVERSITY

We support inclusive, diverse, and equitable conduct of research.

Received: October 25, 2022
Revised: February 27, 2023
Accepted: August 4, 2023
Published: August 28, 2023

REFERENCES

1. Baker, N.R. (2008). Chlorophyll Fluorescence: A Probe of Photosynthesis In Vivo. Annu. Rev. Plant Biol. *59*, 89–113. https://doi.org/10.1146/annurev.arplant.59.032607.092759.
2. Meroni, M., Rossini, M., Guanter, L., Alonso, L., Rascher, U., Colombo, R., and Moreno, J. (2009). Remote sensing of solar-induced chlorophyll fluorescence: Review of methods and applications. Remote Sens. Environ. *113*, 2037–2051. https://doi.org/10.1016/j.rse.2009.05.003.
3. Yang, X., Tang, J., Mustard, J.F., Lee, J.-E., Rossini, M., Joiner, J., Munger, J.W., Kornfeld, A., and Richardson, A.D. (2015). Solar-induced chlorophyll fluorescence that correlates with canopy photosynthesis on diurnal and seasonal scales in a temperate deciduous forest. Geophys. Res. Lett. *42*, 2977–2987. https://doi.org/10.1002/2015GL063201.
4. Ni, Z., Lu, Q., Huo, H., and Zhang, H. (2019). Estimation of chlorophyll fluorescence at different scales: A review. Sensors *19*, 3000. https://doi.org/10.3390/S19133000.
5. Ptushenko, O.S., Ptushenko, V. v, and Solovchenko, A.E. (2020). Life Spectrum of Light as a Determinant of Plant Functioning: A Historical Perspective. https://doi.org/10.3390/life10030025.
6. Evans, J.R. (1999). Leaf anatomy enables more equal access to light and CO2 between chloroplasts. New Phytol. *143*, 93–104. https://doi.org/10.1046/j.1469-8137.1999.00440.x.
7. Qi, Y., Bai, S., Vogelmann, T.C., and Heisler, G.M. (2003). In Penetration of UV-A, UV-B, blue, and red light into leaf tissues of pecan measured by a fiber optic microprobe system, J.R. Slusser, J.R. Herman, and W. Gao, eds., p. 281. https://doi.org/10.1117/12.506629.
8. Rappaport, F., Béal, D., Joliot, A., and Joliot, P. (2007). On the advantages of using green light to study fluorescence yield changes in leaves. Biochim. Biophys. Acta *1767*, 56–65. https://doi.org/10.1016/j.bbabio.2006.10.002.
9. Terashima, I., Fujita, T., Inoue, T., Chow, W.S., and Oguchi, R. (2009). Green Light Drives Leaf Photosynthesis More Efficiently than Red Light in Strong White Light: Revisiting the Enigmatic Question of Why Leaves are Green. Plant Cell Physiol. *50*, 684–697. https://doi.org/10.1093/pcp/pcp034.
10. Sato, Y., Wada, M., and Kadota, A. (2001). Choice of tracks, microtubules and/or actin filaments for chloroplast photo-movement is differentially controlled by phytochrome and a blue light receptor. J. Cell Sci. *114*, 269–279. https://doi.org/10.1242/jcs.114.2.269.
11. Wada, M., Kagawa, T., and Sato, Y. (2003). Chloroplast Movement. Annu. Rev. Plant Biol. *54*, 455–468. https://doi.org/10.1146/annurev.arplant.54.031902.135023.
12. Cazzaniga, S., Dall' Osto, L., Kong, S.-G., Wada, M., and Bassi, R. (2013). Interaction between avoidance of photon absorption, excess energy dissipation and zeaxanthin synthesis against photooxidative stress in Arabidopsis. Plant J. *76*, 568–579. https://doi.org/10.1111/tpj.12314.
13. Schreiber, U. (2004). Pulse-Amplitude-Modulation (PAM) Fluorometry and Saturation Pulse Method: An Overview. In Chlorophyll a Fluorescence. Advances in Photosynthesis and Respiration, G.C. Papageorgiou and Govindjee., eds. (Springer), pp. 279–319. https://doi.org/10.1007/978-1-4020-3218-9_11.
14. Maxwell, K., and Johnson, G.N. (2000). Chlorophyll fluorescence—a practical guide. J. Exp. Bot. *51*, 659–668. https://doi.org/10.1093/jexbot/51.345.659.
15. Ogawa, T., Misumi, M., and Sonoike, K. (2017). Estimation of photosynthesis in cyanobacteria by pulse-amplitude modulation chlorophyll fluorescence: problems and solutions. Photosynth. Res. *133*, 63–73. https://doi.org/10.1007/s11120-017-0367-x.
16. Butler, W.L. (1978). Energy Distribution in the Photochemical Apparatus of Photosynthesis. Annu. Rev. Plant Physiol. *29*, 345–378. https://doi.org/10.1146/annurev.pp.29.060178.002021.
17. Schindelin, J., Arganda-Carreras, I., Frise, E., Kaynig, V., Longair, M., Pietzsch, T., Preibisch, S., Rueden, C., Saalfeld, S., Schmid, B., et al. (2012). Fiji: an open-source platform for biological-image analysis. Nat. Methods *9*, 676–682. https://doi.org/10.1038/nmeth.2019.
18. Uwizeye, C., Decelle, J., Jouneau, P.-H., Flori, S., Gallet, B., Keck, J.-B., Bo, D.D., Moriscot, C., Seydoux, C., Chevalier, F., et al. (2021). Morphological bases of phytoplankton energy management and physiological responses unveiled by 3D subcellular imaging. Nat. Commun. *12*, 1049. https://doi.org/10.1038/s41467-021-21314-0.
19. Finazzi, G., Johnson, G.N., Dallosto, L., Joliot, P., Wollman, F.-A., and Bassi, R. (2004). A zeaxanthin-independent nonphotochemical quenching mechanism localized in the photosystem II core complex. Proc. Natl. Acad. Sci. USA *101*, 12375–12380. https://doi.org/10.1073/pnas.0404798101.
20. Gerotto, C., Alboresi, A., Giacometti, G.M., Bassi, R., and Morosinotto, T. (2012). Coexistence of plant and algal energy dissipation mechanisms in the moss *Physcomitrella patens*. New Phytol. *196*, 763–773. https://doi.org/10.1111/j.1469-8137.2012.04345.x.
21. Baroli, I., and Niyogi, K.K. (2000). Molecular genetics of xanthophyll–dependent photoprotection in green algae and plants. Philos. Trans. R. Soc. Lond. B Biol. Sci. *355*, 1385–1394. https://doi.org/10.1098/rstb.2000.0700.
22. Cove, D. (2005). The Moss *Physcomitrella patens*. Annu. Rev. Genet. *39*, 339–358. https://doi.org/10.1146/annurev.genet.39.073003.110214.
23. Yamashita, H., Sato, Y., Kanegae, T., Kagawa, T., Wada, M., and Kadota, A. (2011). Chloroplast actin filaments organize meshwork on the photorelocated chloroplasts in the moss Physcomitrella patens. Planta *233*, 357–368. https://doi.org/10.1007/s00425-010-1299-2.
24. Michaels, A.F., Caron, D.A., Swanberg, N.R., Howse, F.A., and Michaels, C.M. (1995). Planktonic sarcodines (Acantharia, Radiolaria, Foraminifera) in surface waters near Bermuda: abundance, biomass and vertical flux. J. Plankton Res. *17*, 131–163. https://doi.org/10.1093/plankt/17.1.131.
25. Decelle, J., Probert, I., Bittner, L., Desdevises, Y., Colin, S., de Vargas, C., Galí, M., Simó, R., and Not, F. (2012). An original mode of symbiosis in open ocean plankton. Proc. Natl. Acad. Sci. USA *109*, 18000–18005. https://doi.org/10.1073/pnas.1212303109.
26. de Vargas, C., Audic, S., Henry, N., Decelle, J., Mahé, F., Logares, R., Lara, E., Berney, C., le Bescot, N., Probert, I., et al. (2015). Eukaryotic plankton diversity in the sunlit ocean. Science *348*, 1261605. https://doi.org/10.1126/science.1261605.
27. Guidi, L., Chaffron, S., Bittner, L., Eveillard, D., Larhlimi, A., Roux, S., Darzi, Y., Audic, S., Berline, L., Brum, J., et al. (2016). Plankton networks driving carbon export in the oligotrophic ocean. Nature *532*, 465–470. https://doi.org/10.1038/nature16942.
28. Biard, T., Stemmann, L., Picheral, M., Mayot, N., Vandromme, P., Hauss, H., Gorsky, G., Guidi, L., Kiko, R., and Not, F. (2016). In situ imaging reveals the biomass of giant protists in the global ocean. Nature *532*, 504–507. https://doi.org/10.1038/nature17652.
29. Decelle, J., Stryhanyuk, H., Gallet, B., Veronesi, G., Schmidt, M., Balzano, S., Marro, S., Uwizeye, C., Jouneau, P.-H., Lupette, J., et al. (2019). Algal Remodeling in a Ubiquitous Planktonic Photosymbiosis. Curr. Biol. *29*, 968–978.e4. https://doi.org/10.1016/j.cub.2019.01.073.
30. Uwizeye, C., Mars Brisbin, M., Gallet, B., Chevalier, F., LeKieffre, C., Schieber, N.L., Falconet, D., Wangpraseurt, D., Schertel, L., Stryhanyuk, H., et al. (2021). Cytoklepty in the plankton: A host strategy to optimize

the bioenergetic machinery of endosymbiotic algae. Proc. Natl. Acad. Sci. USA *118*, e2025252118. https://doi.org/10.1073/pnas.2025252118.

31. Allen, A.E., LaRoche, J., Maheswari, U., Lommer, M., Schauer, N., Lopez, P.J., Finazzi, G., Fernie, A.R., and Bowler, C. (2008). Whole-cell response of the pennate diatom *Phaeodactylum tricornutum* to iron starvation. Proc. Natl. Acad. Sci. USA *105*, 10438–10443. https://doi.org/10.1073/pnas.0711370105.

32. Naumann, B., Busch, A., Allmer, J., Ostendorf, E., Zeller, M., Kirchhoff, H., and Hippler, M. (2007). Comparative quantitative proteomics to investigate the remodeling of bioenergetic pathways under iron deficiency in-Chlamydomonas reinhardtii. Proteomics *7*, 3964–3979. https://doi.org/10.1002/pmic.200700407.

33. Crumpton-Taylor, M., Grandison, S., Png, K.M.Y., Bushby, A.J., and Smith, A.M. (2012). Control of Starch Granule Numbers in Arabidopsis Chloroplasts. Plant Physiol. *158*, 905–916. https://doi.org/10.1104/pp.111.186957.

34. Takemura, K., Kamachi, H., Kume, A., Fujita, T., Karahara, I., and Hanba, Y.T. (2017). A hypergravity environment increases chloroplast size, photosynthesis, and plant growth in the moss Physcomitrella patens. J. Plant Res. *130*, 181–192. https://doi.org/10.1007/s10265-016-0879-z.

35. Wuyts, N., Massonnet, C., Dauzat, M., and Granier, C. (2012). Structural assessment of the impact of environmental constraints on Arabidopsis thaliana leaf growth: a 3D approach. Plant Cell Environ. *35*, 1631–1646. https://doi.org/10.1111/j.1365-3040.2012.02514.x.

36. Omasa, K., Konishi, A., Tamura, H., and Hosoi, F. (2009). 3D Confocal Laser Scanning Microscopy for the Analysis of Chlorophyll Fluorescence Parameters of Chloroplasts in Intact Leaf Tissues. Plant Cell Physiol. *50*, 90–105. https://doi.org/10.1093/pcp/pcn174.

37. Tseng, Y.-C., and Chu, S.-W. (2017). High spatio-temporal-resolution detection of chlorophyll fluorescence dynamics from a single chloroplast with confocal imaging fluorometer. Plant Methods *13*, 43. https://doi.org/10.1186/s13007-017-0194-2.

38. Cove, D., Schild, A., Ashton, N.W., and Hartmann, E. (1978). GENETIC AND PHYSIOLOGICAL STUDIES OF THE EFFECT OF LIGHT ON THE DEVELOPMENT OF THE MOSS, PHYSCOMITRELLA PATENS. Annual European Symposium on Photomorphogenesis, 249–254. https://doi.org/10.1016/B978-0-08-022677-4.50026-5.

39. Thelander, M., Olsson, T., and Ronne, H. (2005). Effect of the energy supply on filamentous growth and development in Physcomitrella patens. J. Exp. Bot. *56*, 653–662. https://doi.org/10.1093/jxb/eri040.

40. Xiao, L., Wang, H., Wan, P., Kuang, T., and He, Y. (2011). Genome-wide transcriptome analysis of gametophyte development in Physcomitrella patens. BMC Plant Biol. *11*, 177. https://doi.org/10.1186/1471-2229-11-177.

41. Bhattacharya, D., Archibald, J.M., Weber, A.P.M., and Reyes-Prieto, A. (2007). How do endosymbionts become organelles? Understanding early events in plastid evolution. Bioessays *29*, 1239–1246. https://doi.org/10.1002/bies.20671.

42. Karkar, S., Facchinelli, F., Price, D.C., Weber, A.P.M., and Bhattacharya, D. (2015). Metabolic connectivity as a driver of host and endosymbiont integration. Proc. Natl. Acad. Sci. USA *112*, 10208–10215. https://doi.org/10.1073/pnas.1421375112.

43. Cenci, U., Bhattacharya, D., Weber, A.P.M., Colleoni, C., Subtil, A., and Ball, S.G. (2017). Biotic Host–Pathogen Interactions As Major Drivers of Plastid Endosymbiosis. Trends Plant Sci. *22*, 316–328. https://doi.org/10.1016/j.tplants.2016.12.007.

44. Wright, I.J., Reich, P.B., Westoby, M., Ackerly, D.D., Baruch, Z., Bongers, F., Cavender-Bares, J., Chapin, T., Cornelissen, J.H.C., Diemer, M., et al. (2004). The worldwide leaf economics spectrum. Nature *428*, 821–827. https://doi.org/10.1038/nature02403.

45. Fedorov, A., Beichel, R., Kalpathy-Cramer, J., Finet, J., Fillion-Robin, J.-C., Pujol, S., Bauer, C., Jennings, D., Fennessy, F., Sonka, M., et al. (2012). 3D Slicer as an image computing platform for the Quantitative Imaging Network. Magn. Reson. Imaging *30*, 1323–1341. https://doi.org/10.1016/j.mri.2012.05.001.

46. Ashton, N.W., Grimsley, N.H., and Cove, D.J. (1979). Analysis of gametophytic development in the moss, Physcomitrella patens, using auxin and cytokinin resistant mutants. Planta *144*, 427–435. https://doi.org/10.1007/BF00380118.

47. Carbonera, D., Gerotto, C., Posocco, B., Giacometti, G.M., and Morosinotto, T. (2012). NPQ activation reduces chlorophyll triplet state formation in the moss Physcomitrella patens. Biochim. Biophys. Acta *1817*, 1608–1615. https://doi.org/10.1016/j.bbabio.2012.05.007.

48. Seydoux, C., Storti, M., Giovagnetti, V., Matuszyńska, A., Guglielmino, E., Zhao, X., Giustini, C., Pan, Y., Blommaert, L., Angulo, J., et al. (2022). Impaired photoprotection in *Phaeodactylum tricornutum* KEA3 mutants reveals the proton regulatory circuit of diatoms light acclimation. New Phytol. *234*, 578–591. https://doi.org/10.1111/nph.18003.

CellPress
OPEN ACCESS

STAR★METHODS

KEY RESOURCES TABLE

REAGENT or RESOURCE	SOURCE	IDENTIFIER
Experimental models: Organisms/strains		
Physcomitrium patens Gransden	Gerotto et al.[20]	N/A
Physcomitrium patens lhcsr1-2 KO	Gerotto et al.[20]	N/A
Flaveria robusta	HHU, Dusseldorf	N/A
Flaveria bidentis	HHU, Dusseldorf	N/A
Plantago lanceolata	HHU, Dusseldorf	N/A
Phaeocystis cordata	Decelle et al.[29]	Roscoff Culture Collection RCC1383
Acantharia	Decelle et al.[25] Villefranche-sur-Mer	N/A
Software and algorithms		
ZEISS ZEN 3.0 (blue edition)	Carl Zeiss Microscopy	https://www.zeiss.com/microscopy/fr/produits/logiciel/zeiss-zen-core.html
Fiji	Schindelin et al.[17]	https://ImageJ.net/software/fiji/downloads
OriginPro version 9.0	OriginLab Corporation, Northampton, MA, USA	https://www.originlab.com/
3D Slicer - Slicer 4	Fedorov et al.[45]	https://www.slicer.org/
R software	R Core Team	https://www.R-project.org/.
Custom Code	Clarisse Uwizeye. (2023). compute-NPQ (v.1.0.0)	https://doi.org/10.5281/zenodo.8155231
Other		
LED module	JBeamBio, La Rochelle, France	N/A

RESOURCE AVAILABILITY

Lead contact

Further information and requests for resources and reagents should be direct to and will be fullfilled by the lead contact, Dimitri Tolleter (dtolleter@gmail.com).

Materials availability

This study did not generate new unique reagents.

Data and code availability

- All data reported in this paper will be shared by the lead contact upon request.
- Original code is available at this address Clarisse Uwizeye. (2023). compute-NPQ (v.1.0.0). Zenodo. https://doi.org/10.5281/zenodo.8155231
- Any additional information required to reanalyze the data reported in this paper is available from the lead contact upon request.

EXPERIMENTAL MODEL AND SUBJECT DETAILS

Photosynthetic models used in this study

Physcomitrium patens Gransden wild-type (WT) strain and *lhcsr1-lhcsr2* KO (*lhcsr1-2*)[20] were grown on PpNO3 (3 mM $Ca(NO_3)_2$, 1 mM $MgSO_4$, 50 μM $FeSO_4$, 200 μM KH_2PO_4 pH 7, traces elements)[46] solidified media (0.8% Agar type A suitable for plant cell culture) overlaid with a cellophane filter. Moss tissue was propagated vegetatively by homogenization through a tissue blender and cultivated in axenic condition at 25°C, 40 μmol photons m^2 s^{-1} continuous illumination. 10-days-old moss protonema was employed for confocal microscopy measurement.

Non treated seeds of Flaveria robusta, *Flaveria bidentis* and *Plantago lanceolata* has been sown on soil (Floradur B fin, Soufflet Vigne, Puteaux, France) and grown 3–4 weeks in controlled growth chambers in long day conditions (16 h light/8 h dark) at a PPF of

100 μmol photons m^{-2} s^{-1}. Air temperature was 22°C during the day and 21.0 °C at night. Relative humidity was constant at 70% during the day and night. Young fully developed leaves were chosen for the experiments.

Symbiotic acantharians harboring intracellular microalgal cells (*Phaeocystis cordata*) were gently collected by towing a plankton net of 150 μm in mesh size with a large cod-end (1 L) for 1–2 min in surface waters (Mediterranean Sea, Villefranche-sur-Mer, France). After collection, individual cells were isolated under a binocular with a micropipette.[25] Cells were rapidly transferred to natural seawater and maintained at 20°C and 100 μmol photons m^2 s^{-1} controlled illumination. Samples were imaged within 24 h from sampling time. Cultures of the haptophyte *P. cordata* (the symbiont of Acantharia in the Mediterranean Sea algal,[29] strain RCC1383 from the Roscoff Culture Collection) were maintained at 20°C in K5 culture medium at 100 μmol photons m^{-2} s^{-1}·20°C.

METHOD DETAILS

Sample preparation

P. patens. A small portion (~5 mm diameter) of *P. patens* protonema was endorsed on a 20 × 20 mm coverslip and soaked in 100 μL of water in order to spread the filaments. The coverslip was fixed to the microscopy slide by using a double-side tape of 0.15 mm thickness and finally sealed using VALAP (1:1:1 Vaseline, Lanolin, Paraffin) to prevent water evaporation.

P. cordata and *Acantharians* were settled on a microscopy glass bottom dish (Ibidi, Germany) directly on the microscopy plate to avoid media perturbation.

F. robusta, *F. bidentis* and *P. lanceolata*. 5 × 3 mm sections (longer axis parallel to major leaf veins) were excised using a scalpel. Sections were enclosed between two 20 × 20 mm coverslips allowing to observe both sides of the leaves. A double layer of tape was used to create an enclosure to lodge the leaf section and prevent its crushing. Water was provided to the sample to prevent dehydration during NPQ measurements.

All samples were dark adapted for at least 20 min before introduction in the setup.

Confocal microscope setup

The Zeiss LSM 900 microscope was equipped with continuous light and pulses of strong actinic light, provided by a LED module located in front of the biological sample. The module contains 4 red LEDs (OSRAM, LA W5a.m., λ = 630 nm, Full Width-Half Maximum 18 nm), equipped with a lens to reduce their divergence. The 4 LEDs were oriented at 42°, to focus their light onto the middle of the sample holding slits. The LEDs deliver actinic light, the intensity of which (100 and 200 and 500 μmols photons m^{-2} s^{-1}) is determined by a 3-position switch located on the front face of the control box. Electronic diagrams of the control box are shown Figure S1. Whenever needed, lower actinic radiation levels were obtained by placing a neutral filter (Kodak ND0.3) between the LED array and the sample. This output is connected to the SVB1 Zeiss module, which controls fluorescence acquisition via confocal microscope through the 'experimental design' routine of the ZEN software (version 3.0 Blue edition). The same routine also triggers the switching on of saturating pulses (2000 μmols photons m^{-2} s^{-1}, duration 1.1 s) to measure of Fm and Fm'. Alternatively, saturating pulses can be switched on manually through a button located on the control box. Refer to Table 1 to install the system on another type of confocal microscope platform than the one described above.

Images were acquired with a 20x/0.8 M27 objective (Zeiss) in confocal mode setting with Airyscan 2 detector. This setup has been chosen for fast acquisition without loss of sensitivity, thanks to the use of a collimation optic. Excitation was provided by a blue laser (488 nm) and Chl fluorescence was collected between 650 and 700 nm. Searching for the object under the microscope was conducted under low light intensity conditions (5 μmol photons. m^{-2}. s^{-1} in white light) to minimize any potential irreversible impact on photosynthesis. Similarly, the determination of z-stacks boundaries was performed at an extremely low intensity level (30 times lower than the one utilized during the experiment) to ensure the integrity of the experiment and prevent undesired effects. The initial z position was set as the first slide where the fluorescence of chloroplasts, derived from chlorophyll fluorescence, could be clearly distinguished from the background. The initial z position was set as the first slide where the fluorescence of chloroplasts, derived from chlorophyll fluorescence, could be clearly distinguished from the background.[21,47] A maximum of 25 z-stacks (512 x 512 pixels, z step size 2 μm) were acquired at the fastest speed (pixel dwell time 1.03 μs, with an illumination of 633 ms per frame), to minimise light exposure and therefore reduce the risk of sample photoinhibition during measurements.

To estimate maximum photosynthetic capacity (Y), a series of 10 consecutive images was acquired (experimental time 6.33 s). A saturating pulse was provided by the external LED source after the fourth acquisition (Figure S2A).

In the case of NPQ measurement, appropriate Z-stacks (xyzt experiment) were selected to include entire cells and maintain plastids in the acquisition field. The blue laser intensity was set at the minimum value (0.3% - 220 μJ cm^{-2}) at which PSII fluorescence reached Fm to avoid excess excitation. The delay for two consecutive acquisitions was set to 1 min to prevent photoinhibion due to laser exposure. 3 points were acquired for dark adapted samples, 6 during exposure to actinic red light (LED module) and 6 points again in the dark to follow NPQ relaxation (experimental time 15 min, Figures S2C–S2F). Stability of Fm measurement (and so NPQ) have been checked during the first 2 min of the experiment (in the dark) confirming the relaxed state of the NPQ.

To calculate the ΦPSII parameter, lower intensities of the actinic laser were chosen, in order not to reach Fm, but rather a steady state level Fs. Maximum fluorescence Fm was instead achieved when the laser and the saturating Orange LED were switched on simultaneously.

CellPress
OPEN ACCESS

In Figures 2A and S3B, Chl fluorescence was instead measured with a conventional imaging setup (Speedzen, JBeamBio, France) described *e.g.*, in Seydoux et al.[48]

QUANTIFICATION AND STATISTICAL ANALYSIS

PSII yield and NPQ calculation

Experimental files were imported to Fiji,[17] using the ''sum slices'' routine and we transform xyzt files into xyt to calculate a time course of fluorescence changes in case of 3D acquisitions. Regions Of Interest (ROIs) were drawn on the Z project image to select single chloroplasts, cells or whole tissues or non-fluorescent regions that were used to estimate the background level (Figure S2A). 'Mean gray value' of fluorescence was quantified for each single ROI and time. Background was subtracted to raw values for each time point. Numeric fluorescence values were imported to Origin software (Microcal, USA) to calculate PSII related parameters. In the case of PSII capacity (Y), F′ value is the mean value for dark adapted samples measured with non-saturating laser light. Fm is instead the maximum value of the fluorescence obtained during exposition to a saturating pulse (Figures S2A and S2B). For NPQ, Fm is the mean value for dark adapted plant exposed to saturating laser intensity; Fm' is the maximum fluorescence value samples exposed to actinic light or during the dark relaxation time (Figure S2B). In the case of acantharians (Figure 4), Fs is the fluorescence measured in the presence of the non-saturating blue laser, while Fm is the fluorescence value achieved in the presence of the laser plus the saturating value of the orange LED.

3D reconstruction and fluorescence integration

Image processing was done adapting a pipeline previously developed for 3D reconstruction based on electron microscopy stacks.[18] Briefly, confocal images were pre-processed using a non-linear median filter (from Fiji) that preserves the edges. This is an essential prerequisite to calculate object volumes (see *e.g.*, Figure 4B). We used 3DSlicer to perform semi-automatic segmentation.[45] To calculate the volume of a reconstructed 3D model, we multiplied the number of voxels (volumetric picture elements) in the object by the size of the voxel: VI = (number of voxels in the object I) × (size of the voxel Vi)

To calculate the fluorescence of each object in a confocal image, images were segmented to obtain the location of a given ROI). Data were used to calculate the sum of the voxel values of a given ROI, and therefore the mean fluorescence value of a given plastid or cell. In the case of *Phaeocystis cordata* photosymbiotic cells, where single plastids could not be imaged because of their small size, fluorescence parameters were calculated on cell sections, which we approximated with a circle (red circle in Figures 4E and 4F). To assess possible heterogeneous responses, we scanned fluorescence values along the radius. We then interpolated the corresponding fluorescence intensity to calculate fluorescence parameters (Fm, Fm'), and therefore NPQ, as a function of the angle. At least ten sections (*i.e.*, 10 μm) were scanned for every cells to assess reproducibility.

Principal components analysis (PCA)

We performed PCA considering six observed variables: NPQav, NPQmax, Decay, Induction, Fmax and Area Fraction. The variables used here were calculated in automatized way from NPQ data. NPQav is the mean NPQ when a cell is exposed to light while NPQmax is the maximum of NPQ reached during the light exposition. Decay and Induction are the relaxation and induction rate of NPQ evaluated from the slope of NPQ changes during the first 2 min of the light to dark and the dark to light transitions, respectively. Area Fraction is the area percentage occupied by the chloroplast inside a cell. Fmax is the ''Mean gray value'' of fluorescence of a given cell chloroplasts for dark adapted samples (Fm).

Variables were measured in 175 cells from *P. patens* chloronema and caulonema in the WT or the *lhcsr1-2* KO strains. The type of cells (mutant/WT or chloronema/caulonema) did not play a role in the determination of the component and that they are used after to characterise the possible biological role of the components. All data are normalized by subtraction of the mean and division by the standard deviation so that the singular values decomposition is done on the correlation matrix of the data (Table S1).

To represent the distribution of these normalized dimensional data for the 175 images, the direction (a 6-dimensional vector) giving the largest possible variance of the distribution was selected as the direction for the first principal component. Then, we selected the direction orthogonal to the previous one(s) giving the largest possible variance of the distribution as the direction for the second principal component. By repeating this procedure automatically, we identify vectors representing the scatter of the distribution from major ones to minor ones (Table S2). Based on singular values decomposition, PCA is a principal axis rotation of the original variables that preserves the variation in the data. Therefore, the total variance of the original variables is equal to the total variance of the principal components. The principal component coefficients correspond to the percentage of explained variance. Statistical analysis was done with the R software (http://www.R-project.org). The table of the original observed variables used to construct the six components (Table S3) provides the interpretation of the components.

Cell Reports Methods, Volume *3*

Supplemental information

Tailoring confocal microscopy

for real-time analysis of photosynthesis

at single-cell resolution

Mattia Storti, Haythem Hsine, Clarisse Uwizeye, Olivier Bastien, Daniel P. Yee, Fabien Chevalier, Johan Decelle, Cécile Giustini, Daniel Béal, Gilles Curien, Giovanni Finazzi, and Dimitri Tolleter

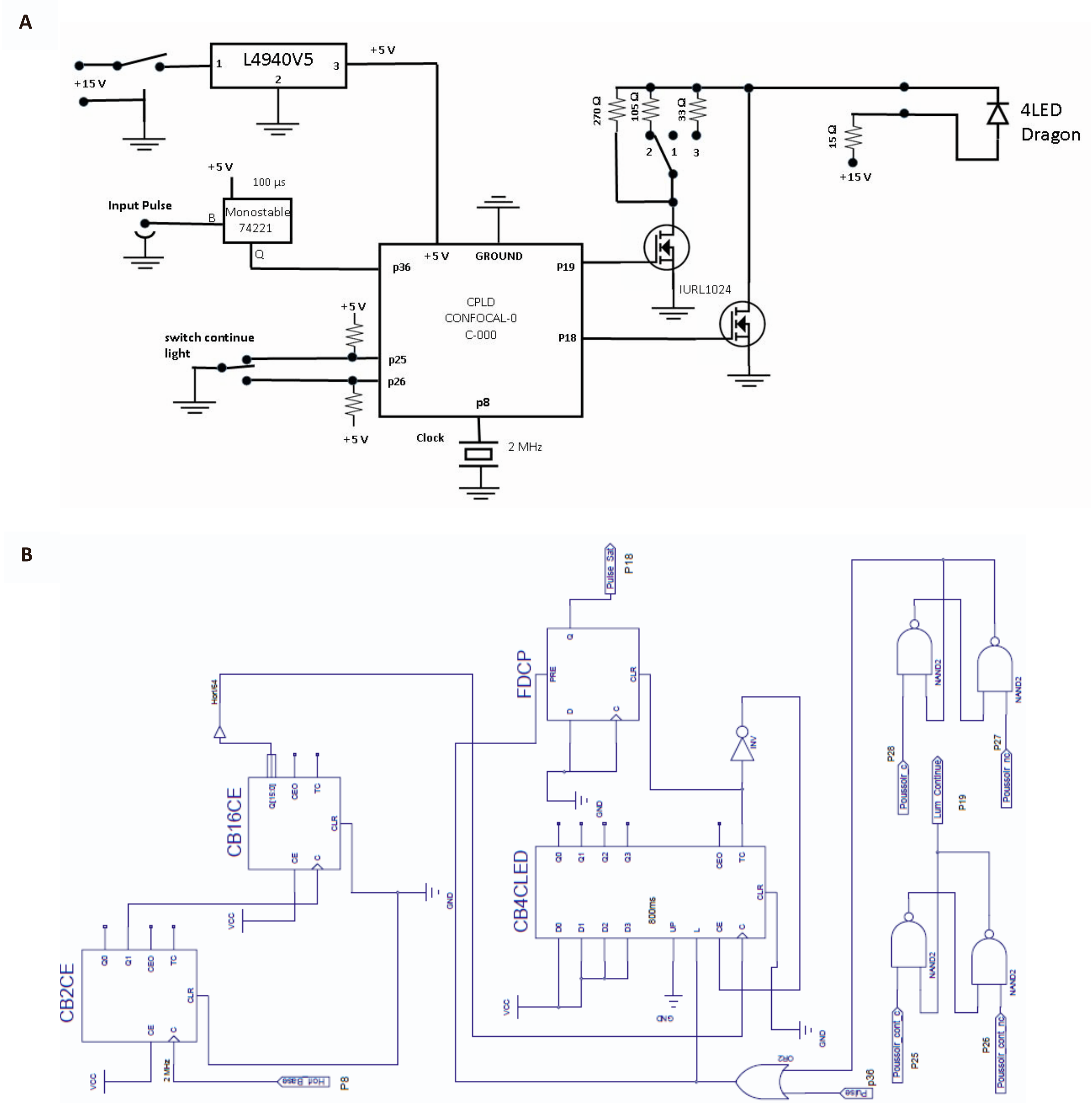

Figure S1 (related to Figure 1): Electronic scheme of the control box. (A), general scheme, **(B)**, details of the CPLD**.**

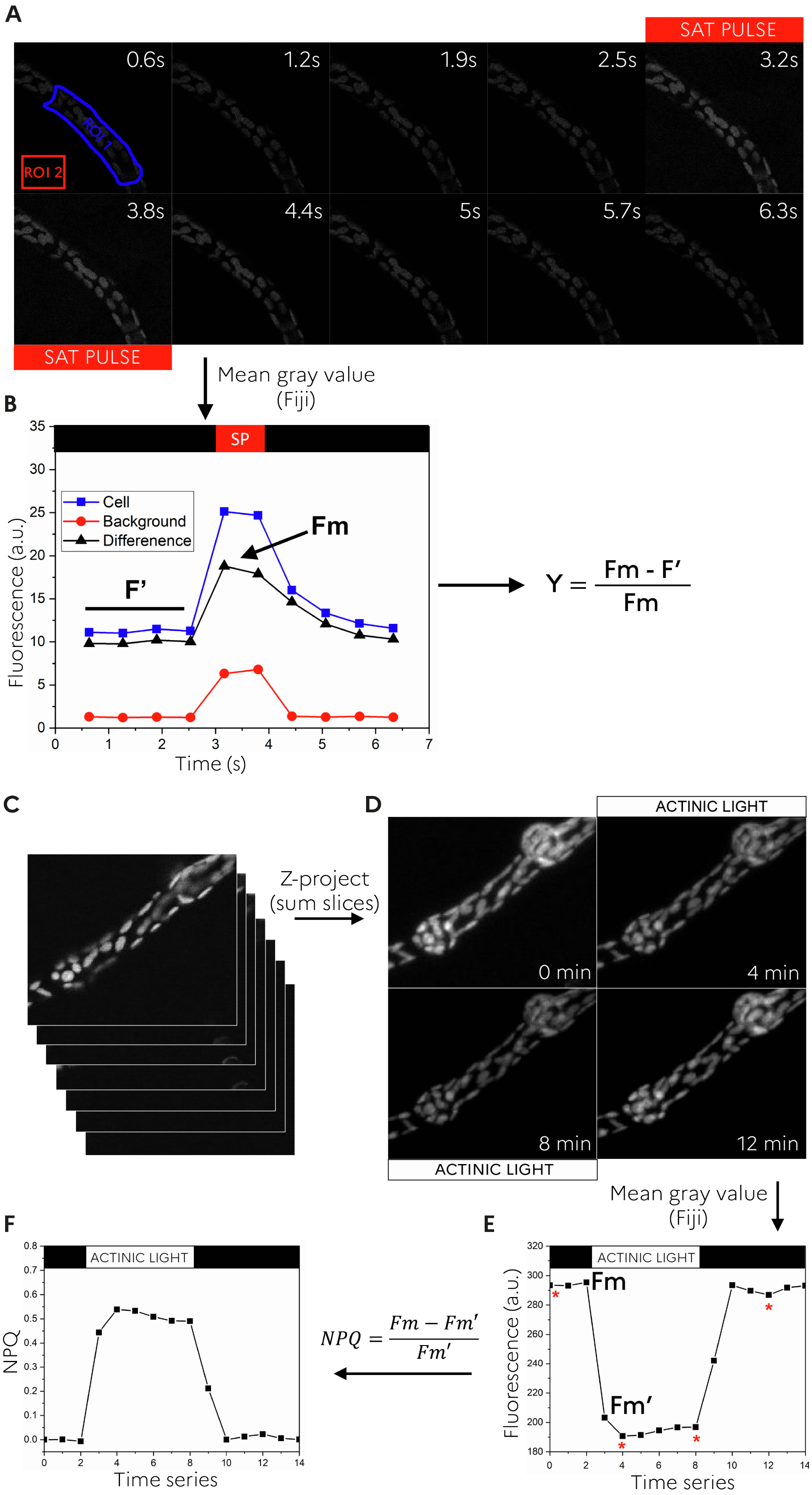

Figure S2 (related to Figure 2): Analysis of confocal images with Fiji. (A), To measure the 'Y' parameters (PSII yield) we calculated the "mean gray value" of ROIs (ROI 1, blue: *P. patens* cell; ROI 2, red background). **(B)**, Background fluorescence (red), which is altered by external light source (Saturating pulse, SP, red bar) was subtracted from the sample fluorescence (blue) value to obtain the real fluorescence value (difference, black). By plotting the results of this calculation for sequential acquisitions, we obtained a time series, which we used to calculate Y values. For NPQ experiments, xyzt files **(C)** were converted to xyt files **(D)** using the "sum slices" function of Fiji. Images were analysed as in (A) to obtain fluorescence **(E)**. Fm is the fluorescence value of the dark-adapted sample (top black bar) while Fm' is the fluorescence value measured during exposition to actinic light (top white bar). Both values are needed to calculate NPQ **(F)**. Red asterisks in (E) represent the fluorescence values calculated from the images represented in panel D.

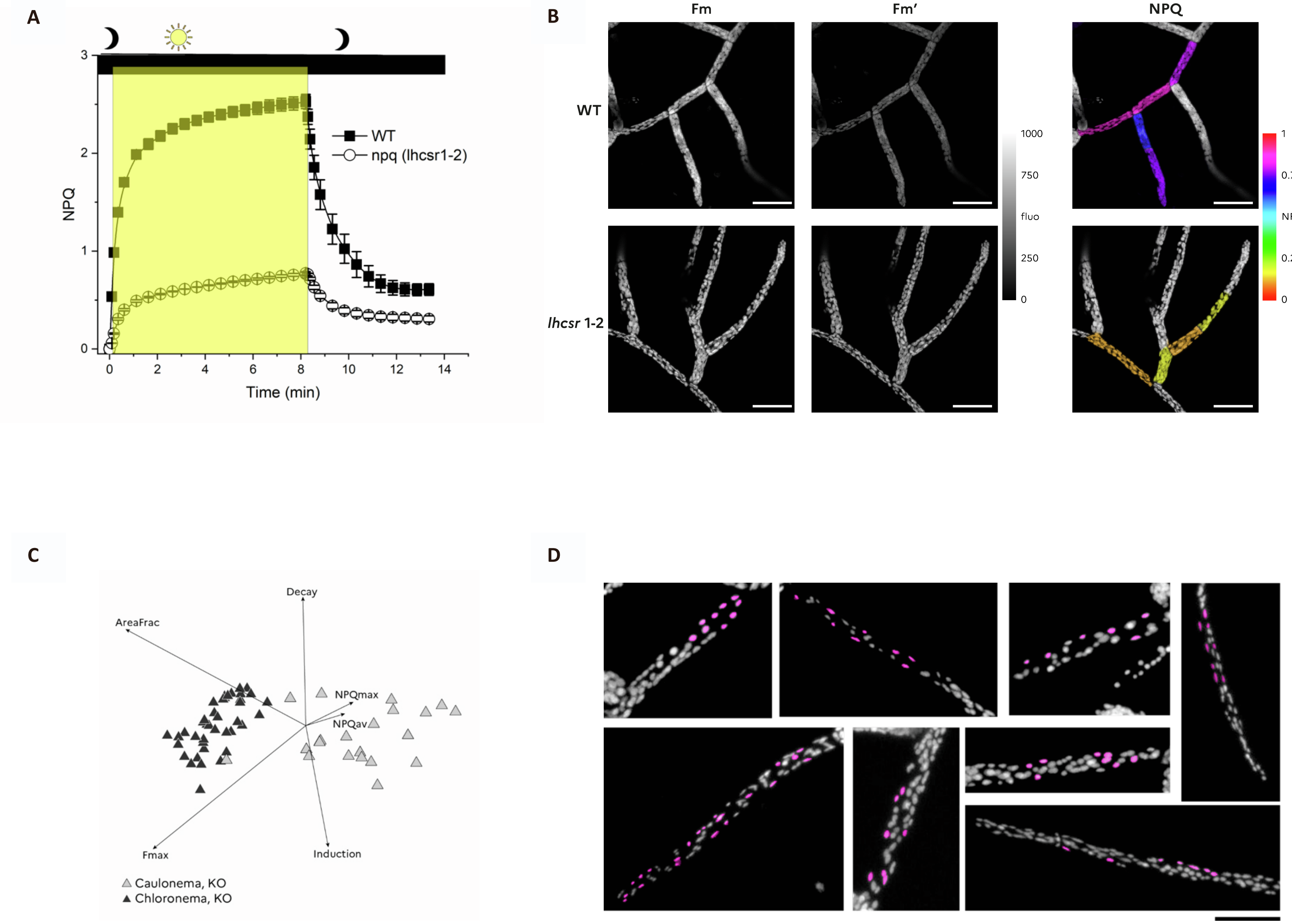

Figure S3 (related to figure 2-3): NPQ analysis of *P. patens* cells. (A), NPQ in *P. patens*, measured with a chlorophyll fluorescence imaging camera. WT (black squares) and *lhcsr1-2 (empty circles) P. patens* protonema were exposed to saturating light intensity (yellow overlay, 500 µmols photons m^{-2} s^{-1}) for 8 min before measuring photosynthetic parameters using a speedzen Chl fluorescence imaging setup (JBeambio, France).[47] Same symbols and box colours as in Fig 2D. **(B)**, Comparison between WT cells and mutant cells with reduced NPQ capacity (*lhcsr1-2* KO) Fm and Fm' represent the maximum fluorescence under dark condition and under actinic light respectively (grayscale). NPQ was calculated from Fm and Fm' and is visualised by artificial colour only for the analysed cells of the WT and mutant protonema. Scale bars 50µm. **(C)**, PCA analysis of NPQ features in the *P. patens lhcsr1-2* KO-mutant: second and third components**.** Second and third components of a PCA realised on 63 *P. patens lhcrs1/2* KO cells (solid symbols chloronema, open symbols: caulonema). The first two components represent roughly 88% of the variance and the first three components represents more than 94% of the variance (Supplementary Table 2). **(D)**, NPQ analysis of *P. Patens* single chloroplasts. Among the numerous plastids of *P. patens* protonema, some single chloroplasts (highlighted in pink) were selected because they never overlap with each other during the confocal microscope acquisition. Chloroplasts movements were tracked manually and fluorescence values were calculated in the dark and after 6 min of light exposure (500 µmol photons m^{-2} s^{-1}). Scale bar: 50 µm.

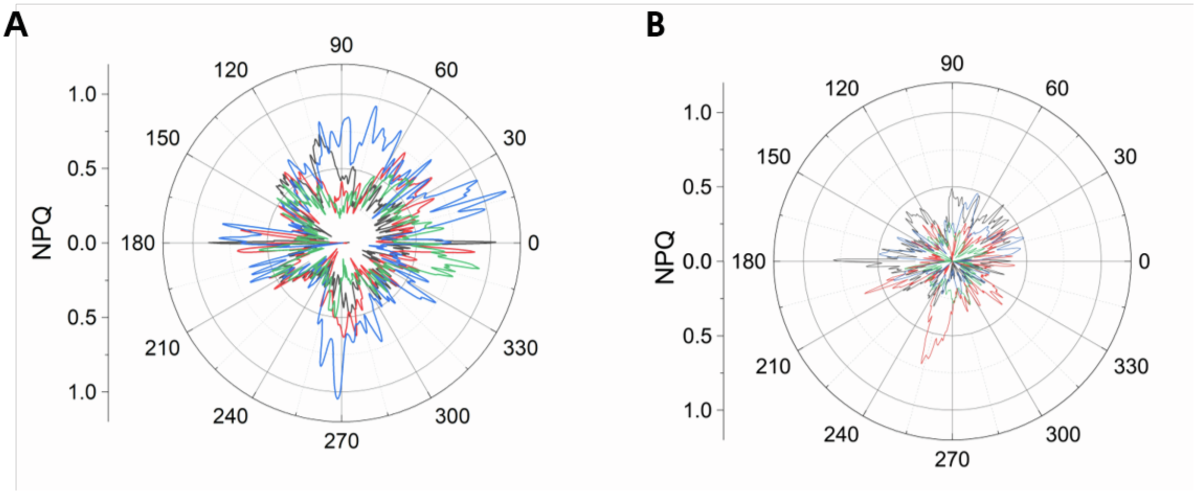

Figure S4 (related to Figure 4): subcellular analysis of NPQ in acantharians. Radar plot of the NPQ in large **(A)** and small **(B)** symbiotic microalgae. NPQ was calculated from images acquired in the dark and after exposure to actinic light (500 µmol photons m^{-2} s^{-1}) for 15 minutes. Different colours represent different phaeocystis cells.

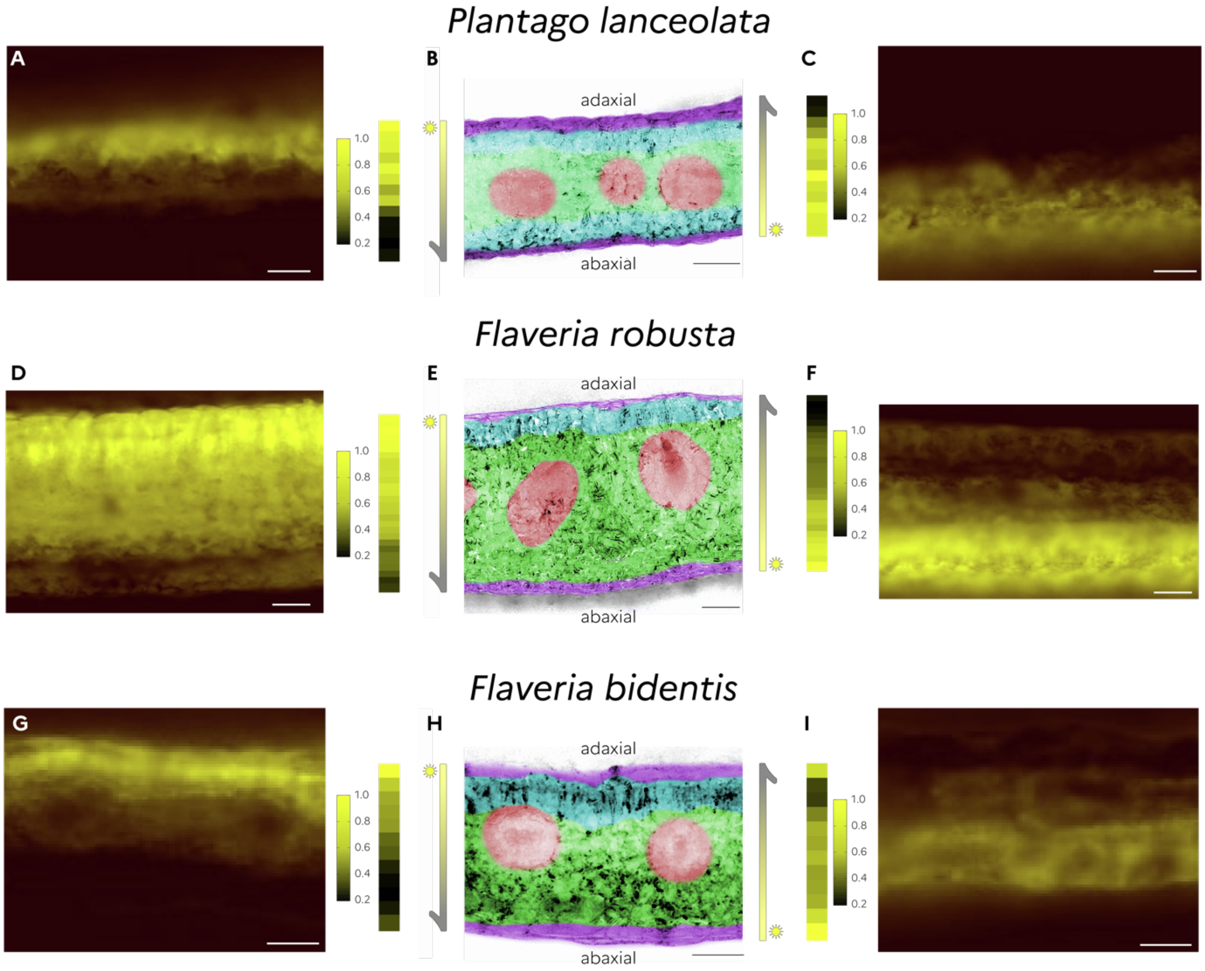

Figure S5 (related to Figure 5): Light gradients inside leaves. Light gradients are visualised by the difference in chlorophyll fluorescence (figured as yellow in artificial colour) intensity upon illumination in the adaxial to abaxial side (bottom to top –panels **A**, **D** and **G**) and abaxial to adaxial (bottom to top –panels **C**, **F** and **I**) for a representative cross section of leaves with different anatomies (panels **B**, **E** and **H** Scale bar : 100µm). The following colours were used to highlight the different leaf tissues: purple: epidermis; blue: palisade parenchyma; green: spongy parenchyma; red: vascular tissue

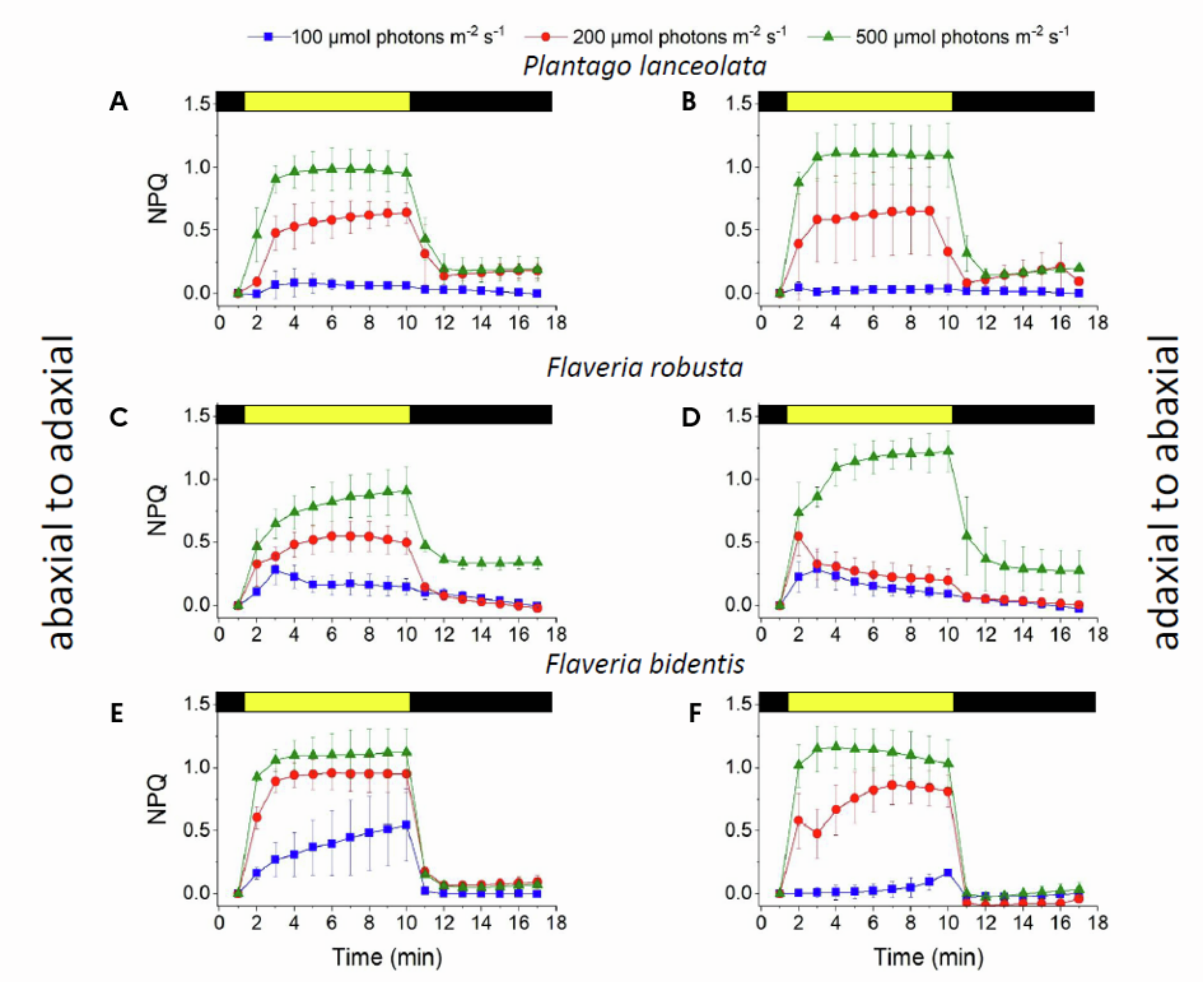

Figure S6 (related to Figure 5): NPQ features are modulated by leaves architectures. NPQ measured upon illumination in the adaxial to abaxial side (top to bottom –panels **A**, **C** and **E**) and abaxial to adaxial (bottom to top – panels **B**, **D** and **F**) for three leaves with different anatomies as presented in Fig. 4. Shown are means ± SDs (n=3)

	Comp. 1	Comp. 2	Comp. 3	Comp. 4	Comp. 5	Comp. 6
Standard deviation	1.9182243	1.2630922	0.60785746	0.45836413	0.35531842	0.138470303
Proportion of Variance	0.6132641	0.2659003	0.06158178	0.03501628	0.02104186	0.003195671
Cumulative Proportion	0.6132641	0.8791644	0.94074619	0.97576247	0.99680433	1.000000000

Supplementary Table 1 (related to Figure 3): Correlation table of the six observed variables

	NPQmax	NPQav	Decay	Induction	AreaFrac	Fmax
NPQmax	1.00000000	0.97681818	0.8537735	0.812678625	-0.070494246	0.2514870
NPQav	0.97681818	1.00000000	0.8392191	0.837617127	-0.020657062	0.2833078
Decay	0.85377353	0.83921910	1.0000000	0.701930935	0.195843670	0.3648589
Induction	0.81267862	0.83761713	0.7019309	1.000000000	0.009117372	0.3559048
AreaFrac	-0.07049425	-0.02065706	0.1958437	0.009117372	1.000000000	0.6719592
Fmax	0.25148703	0.28330778	0.3648589	0.355904772	0.671959243	1.0000000

Supplementary Table 2 (related to Figure 3): Principal Component Analysis results.

	1st Comp	2nd Comp	3rd Comp	4th Comp	5th Comp	6th Comp
NPQmax	24.4496826	3.7675641	0.9885236	3.6558842	14.9788645	52.15948102
NPQav	24.8978544	2.5525258	0.2549017	0.2827022	25.9588445	46.05317140
Decay	22.5153461	0.0140642	29.3140005	3.7408834	43.5351179	0.88058787
Induction	21.4494317	0.8431956	26.1007806	42.9901977	8.2970322	0.31936221
AreaFrac	0.6559251	53.9600310	16.4085692	21.8731751	6.5749696	0.52732993
Fmax	6.0317601	38.8626193	26.9332244	27.4571575	0.6551713	0.06006757

Supplementary Table 3 (related to Figure 3): Contribution table of the six observed variables to the construction of the six components. For example, the first component is constructed with a contribution of NPQmax with 24.5 %, NPQav with 24.9%, Decay with 22.5% and Induction with 21.5%
